# Dorsal prefrontal and premotor cortex of the ferret as defined by distinctive patterns of thalamo-cortical projections

**DOI:** 10.1007/s00429-020-02086-7

**Published:** 2020-05-26

**Authors:** Susanne Radtke-Schuller, Stephen M. Town, Pingbo Yin, Diego Elgueda, Gerd Schuller, Jennifer K. Bizley, Shihab A. Shamma, Jonathan B. Fritz

**Affiliations:** 1 Institute for Systems Research, University of Maryland, College Park, MD 20742 USA; 2 Department of Psychiatry, University of North Carolina at Chapel Hill, Chapel Hill, NC 27599 USA; 3 Faculty of Brain Sciences, UCL Ear Institute, University College London, London, WC1X 8EE UK; 4 Department of Animal Pathology, Faculty of Veterinary and Animal Sciences, University of Chile, Santiago, Chile; 5 Munich Center for Neuroscience, Ludwig Maximilian University, Planegg-Martinsried, 82152 Munich, Germany; 6 Department of Cognitive Studies, École Normale Supérieure, 75005 Paris, France; 7 Center for Neural Science, New York University, New York City, NY 10003 USA

**Keywords:** Carnivores, Frontal cortex, Connectivity, Tracing

## Abstract

Recent studies of the neurobiology of the dorsal frontal cortex (FC) of the ferret have illuminated its key role in the attention network, top-down cognitive control of sensory processing, and goal directed behavior. To elucidate the neuroanatomical regions of the dorsal FC, and delineate the boundary between premotor cortex (PMC) and dorsal prefrontal cortex (dPFC), we placed retrograde tracers in adult ferret dorsal FC anterior to primary motor cortex and analyzed thalamo-cortical connectivity. Cyto- and myeloarchitectural differences across dorsal FC and the distinctive projection patterns from thalamic nuclei, especially from the subnuclei of the medial dorsal (MD) nucleus and the ventral thalamic nuclear group, make it possible to clearly differentiate three separate dorsal FC fields anterior to primary motor cortex: polar dPFC (dPFCpol), dPFC, and PMC. Based on the thalamic connectivity, there is a striking similarity of the ferret’s dorsal FC fields with other species. This possible homology opens up new questions for future comparative neuroanatomical and functional studies.

## Introduction

The ferret is a promising animal model for exploring the neurobiology of the frontal cortex (FC). It has gained increasing interest as a result of recent studies on the role of the dorsal FC in top-down cognitive control of sensory processing (Fritz et al. 2010; Francis et al. 2018; Bimbard et al. 2018; Elgueda et al. 2019), in goal-directed behavior (Zhou et al. 2016), in the interactions in the frontoparietal attention network (Sellers et al. 2016), and as a model system to study the effects of anesthesia (Sellers et al. 2013, 2015; Wollstadt et al. 2017). Moreover, in vitro studies of FC functional microcircuitry have also been carried out in the ferret (Krimer and Goldman-Rakic 2001; McCormick et al. 2003; Shu et al. 2006, 2007; Wang et al. 2006; Winograd et al. 2008) and have been recently developed as an in vitro model of schizophrenia (Rebollo et al. 2018).

However, there have hitherto been few neuroanatomical studies of the ferret FC to keep pace with advances in neurophysiological research. The extent of the ferret prefrontal cortex (PFC) as a whole was previously explored in a comprehensive connectivity study (Duque and McCormick 2010), and the ferret’s FC subdivisions have been delineated based on cyto- and myeloarchitectural criteria (Radtke-Schuller 2018). Cyto- and myeloarchitectural criteria, however, are not sufficient for defining the ferret FC subdivisions in comparison with other species. Hence, the present study focuses on the thalamic connectivity of the dorsal FC fields of the ferret.

Anatomically, the frontal lobe was originally defined by Brodmann (1909) as the cortex anterior to the central fissure in humans. It is composed of the agranular motor and premotor cortex (“regio praecentralis”) and the anterior bordering granular “regio frontalis”, which later was named “prefrontal cortex” in humans and in non-human primates. These two criteria for PFC, frontal position and the presence of a granular layer, initially seemed sufficient to define and localize PFC in primates.

**Fig. 1:**
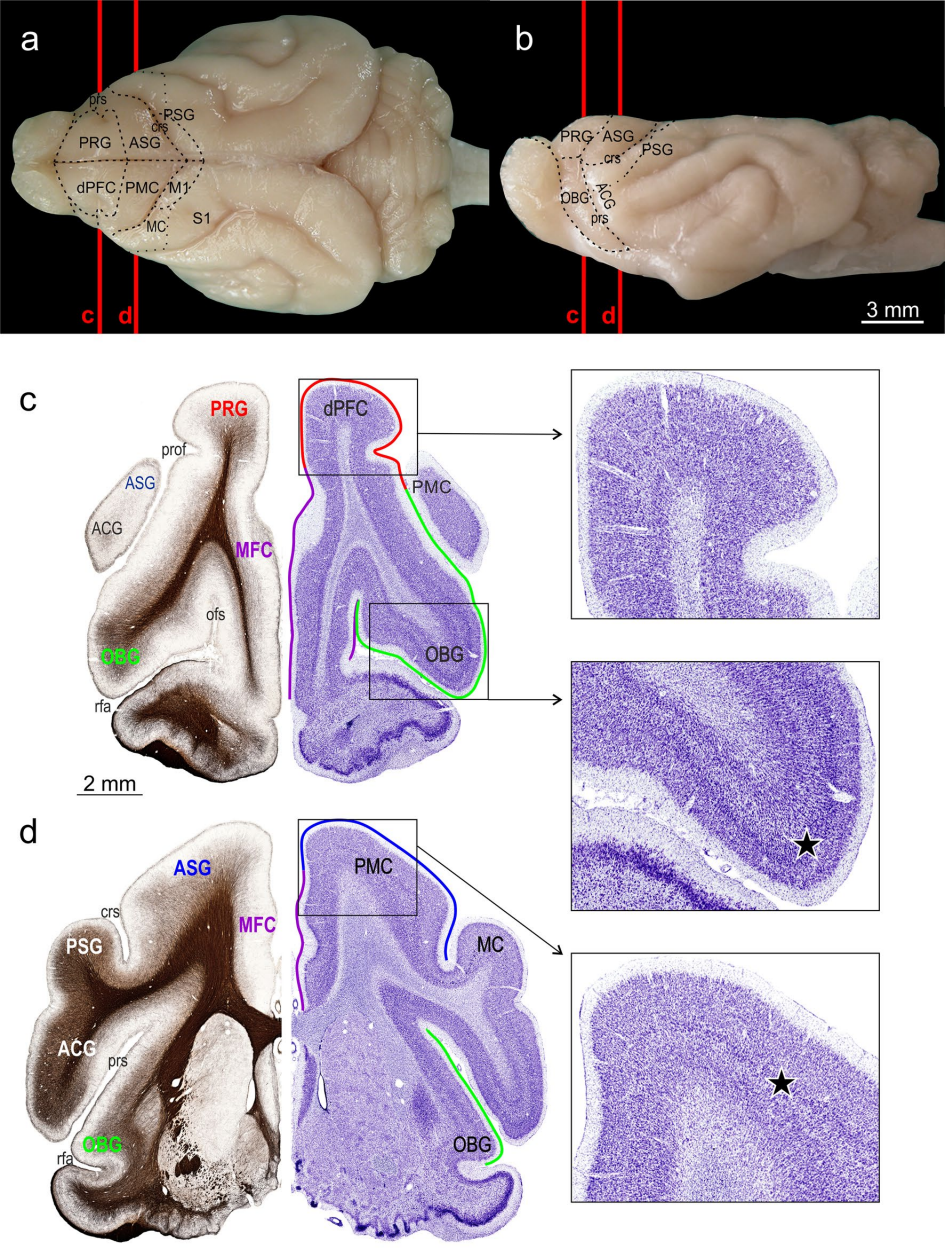
Macroscopic and microscopic anatomy of the ferret’s dorsal FC subdivisions in top view (**a**) and side view (**b**) of the brain. The gyri and sulci are delineated on the right hemisphere of the top view and on the side view. Cortical subdivisions are depicted on the left hemisphere of the top view. The red vertical lines indicate the rostro-caudal levels of frontal sections that are represented in the collages shown in **c**, **d**. The composite frontal sections consist of a Nissl stained semi-section on the right and the neighboring mirrored myelin-stained semi-section on the left. Gyri, sulci, and functional field names are labeled. The colored outlines in the right semi-sections roughly indicate the extension of the gyri labeled with the corresponding color on the left. Scale bar in c also applies to (**d**). The enlargements from (**c**, **d**) demonstrate the different characteristic layering patterns in dPFC, OBG, and PMC, respectively. The cortex of dPFC appears unstructured in comparison to the clear lamination of OBG and the light lamination of PMC. Stars in the insets point to the intensely stained layer III in OBG and upper layer V in PMC. *ACG* anterior composite gyrus, *ASG* anterior sigmoid gyrus, *crs* cruciate sulcus, *dPFC* dorsal prefrontal cortex, *M1* primary motor cortex, *MC* motor cortex, *MFC* medial frontal cortex, *OBG* orbital gyrus, *ofs* olfactory sulcus, *PMC* premotor cortex, *PRG* proreal gyrus, *prof* proreal fissure, *prs* presylvian sulcus, *PSG* posterior sigmoid gyrus, *rfa* rhinal fissure anterior part, *S1* primary somatosensory cortex

In the ferret, there are no superficial landmarks of cortical folding that delimit the region comprising the motor areas. However, the primary motor cortex with its band of very large pyramidal cells in layer V is easily recognized at a microscopic level. The anterior adjacent premotor cortex (PMC) is separated from the primary motor cortex by the cruciate sulcus (Fig. 1). The size of pyramidal cells within PMC gradually declines towards the border with the dorsal PFC (dPFC) in the ferret (Radtke-Schuller 2018, plates 8–10) which is consistent with a similar pattern that has also been described in other mammalian species (primate, cat, dog Walker 1940; Akert 1964; Akert and Hartmann-von Monakow 1980; Rajkowska and Kosmal 1988).

However, in most non-primate mammals including the ferret, the FC does not possess a clear granular layer and, therefore, Brodmann’s definition of PFC is not applicable in this respect for these species. Another possible approach to PFC definition was based on the observation that the strongest subcortical input to PFC derives from the mediodorsal thalamic nucleus (MD). Thus, instead of using Brodmann’s criteria, Rose and Woolsey (1948) proposed that PFC should be defined as the cortical projection field of MD. This suggestion offered a way out of the dilemma of how to define PFC in non-primate mammals with agranular frontal association cortex. Based on this definition, PFC was accordingly localized in many species (for review, see Fuster 2015). Along the same lines, the ferret’s PFC was described based on strong reciprocal connections with MD (Duque and McCormick, 2010).

Although afferent projections from MD are a necessary defining characteristic of PFC, it was later shown that this is not a sufficient condition to define PFC. Neither of the two assumptions of Rose and Woolsey, that MD only projects to PFC, or that MD is the only input to PFC, proved to be true. To establish what can be considered equivalent prefrontal regions between different species is still a major challenge, as can be best seen from the ongoing debate on rodent PFC (for comprehensive reviews on the PFC debate, see (Reep 1984; Preuss 1995; Uylings et al. 2003; Wise 2008; Fuster 2015; Carlen 2017).

It has been shown in many species that different MD subdivisions are preferentially interconnected with distinct FC areas (e.g., primate, cat, dog Akert 1964; cat: Markowitsch et al. 1978; dog: Kosmal 1981a; rat: Ray and Price 1992; macaque monkey: Ray and Price 1993) and the identification of input sources to the ferret’s dorsal FC fields allows for a neuroanatomy-based interspecies comparison. Moreover, in a complementary approach, the other thalamic sources that project to FC can also be considered and compared with those observed in other species. The nuclei of the ventral thalamic group (VNG) are of special interest for the differentiation between dPFC and PMC, as they show a characteristic cortical projection pattern for the different FC fields in carnivores (e.g., dog: Kosmal 1981b) and non-human primates (for review, see Jones 1985).

The present study focuses on the dorsal FC fields of the ferret with the primary aim to substantiate the definition and delineation of dPFC and PMC. Injections of retrograde tracers into the dorsal FC fields were used to identify their thalamic afferent connections. The study also addresses the question as to whether the polar region of the ferret’s dPFC might constitute a separate field (dPFCpol).

## Materials and Methods

Ten healthy adult ferrets (*Mustela putorius furo*; male and female, all older than 4 months) were used in this neuroanatomical study. Experiments were performed either at the University of Maryland (UMD) (cases M1406FR/FE, M1502FE/FR, M1005, M1002L/R, M1410FR, M1505FR/FE) or at University College London (UCL) (cases F1508, F1503, F1505, F1515). The Maryland research was approved by the UMD IACUC (Institutional Animal Care and Use Committee) and conformed to standards specified by the National Institutes of Health. The research at UCL was approved by the local animal care committee at UCL and the Royal Veterinary College and authorized by the UK Home Office.

Neural tracers were injected into different parts of the FC using stereotaxic procedures (Bizley et al. 2015; Radtke-Schuller 2018; Elgueda et al. 2019). In six animals, single tracer injections were made, and in four cases, two injections were placed. The tracers used in the experiments performed at UCL were 1% CTB conjugated to TRITC (cholera toxin B subunit-tetramethylrhodamine B isothiocyanate conjugate (CTB TRITC) from List Biological Laboratories, Campbell, CA). The tracers used in the experiments at UMD were 10% Fluoro-Ruby (FR) (dextran, tetramethylrhodamine, lysine fixable, 10,000 MW; Molecular Probes Inc., Eugene, OR, USA), 10% Fluoro-Emerald (FE) (dextran, fluorescein, lysine fixable, 10,000 MW, anionic; Molecular Probes Inc., Eugene, OR, USA) and solid WGA-HRP (wheat germ agglutinated horseradish peroxidase; Sigma-Aldrich). Details of the tracers used in each FC region and the number of injections indicated by delineations in the overview image are given in the table of (Fig. 4). The use of different tracers helps to avoid the limitations associated with any individual tracer and represents a consolidating validation of results, as long as the results are consistent.

We note that although tracer injections were made in both hemispheres, in all figures, the sections and maps are illustrated as projections on the left hemisphere, to facilitate comparisons between cases.

### Surgical procedure

The surgical procedures have been described in detail previously (UCL cases Bizley et al. 2015; UMD cases Elgueda et al. 2019). Although the procedures were similar, there were differences in the anesthetics used for the surgical procedures followed at UCL and UMD, and other minor differences, which are described below.

UCL cases: anesthesia was induced by a single dose of a mixture of Ketamine (5 mg/kg) and Diazepam (2 mg/kg) and maintained with 1–2% Isoflurane throughout surgery. Animals were treated with pre-operative and post-operative analgesia (Buprenorphine, 0.01–0.03 mg/kg), anti-inflammatories (Loxicam, 0.05 mg/kg), and prophylactic antibiotics (Amoxycare LA, 15 mg/kg). A single dose of atropine (Atrocare (0.006 mg/kg) was provided to minimize secretions in the respiratory tract. During anesthesia, ECG, oxygenation, end-tidal CO_2_ and body temperature were monitored. Post-operative analgesia (Buprenorphine, 0.01–0.03 mg/kg) and anti-inflammatories (Loxicam, 0.05 mg/kg) were provided for 5 days post-surgery.

UMD cases: ferrets were anesthetized with a combination of Ketamine (35 mg/kg IM) and Dexmedetomidine (0.03 mg/kg SC) for induction, and deep levels of anesthesia were maintained with 1 − 2% Isoflurane throughout the surgery. Dexamethasone was administered immediately before surgery to avoid possible cerebral edema. Animals were also medicated with atropine sulfate (0.05 mg/kg SC) to control salivation and to stabilize heart and respiratory rates. During surgery, ECG, pulse and blood oxygenation were monitored, and rectal temperature was maintained at 38 °C. Following surgery, antibiotics (Cefazolin, 25 mg/kg SC) and analgesics (Dexamethasone 2 mg/kg SC and Flunixin Meglumine 0.3 mg/kg SC) were administered.

In both labs, after reaching a deep plane of anesthesia, the animal was mounted in a stereotaxic frame fitted with ear bars, bite bar, and nose clamp, and the head was stabilized for surgery. A midline incision was made in the scalp and the temporal muscle was retracted from the midline to expose the skull. After local application of Marcaine (bupivacaine) (UCL), the targeted area of FC was exposed by a craniotomy and a small opening was made in the dura and pia mater. In some cases (M1406FR/FE, M1502FE/FR, M1505FR/FE), neurophysiological recordings of cortical responses were performed prior to tracer injections in the same areas. The results of these recordings will be reported separately. In all cases, tracers were injected and the exact areas involved in each tracer injection were subsequently determined based on histological examination (see below). FR and FE were injected by pressure with a nano-injector (Nanoject II, Univentor syringe pump and Hamilton syringes) at UMD and CTB with a UMP3 UltraMicroPump (World Precision Instruments) at UCL. WGA-HRP tracer was placed as a small, solid bead. For the application, crystals of WGA-HRP were taken up with the moistened fine tip of a glass micropipette, where the crystals dissolved and dried forming a solid WGA-HRP bead at the micropipette tip. This procedure was repeated by taking up more crystal with the moistened WGA-HRP bead until a bead diameter of 600–700 µm was reached. The glass micropipette was then lowered into the brain, and positioned and left in place for a duration of 15 min to allow for complete dissolution of the WGA-HRP bead.

Injection depth was chosen based on the known cortical thickness of the to-be-injected area, typically 700–900 µm from the cortical surface, targeting layers III-V. Once the injections were complete, the micropipette was left in place for 10 min before being withdrawn. Following removal of the micropipette, the dura was folded back in place, and the piece of cranium, that had previously been removed for the craniotomy, was replaced and secured with Kwik-Sil silicone (World Precision Instruments). The temporal muscle was repositioned over the skull and attached to adjacent musculature and the scalp margins were sutured together.

After a survival time of 2–5 weeks for CTB injections, 10 days for FR/FE injections, and 2 days for WGA-HRP, the animals were given a terminal overdose of Euthatal^®^ (2 ml of 200 mg/ml of pentobarbital sodium) and then transcardially perfused. In the perfusion procedure, after an initial injection of 500 IU of heparin directly into the left ventricle, the blood vessels were flushed with 250 ml of 0.9% phosphate-buffered saline, followed by 750 ml of fresh 4% paraformaldehyde in 0.1 M phosphate buffer at pH 7.4. The brain was removed from the skull, maintained in the same fixative at 4 °C for several hours up to overnight (12–15 h), and then immersed in a 30% sucrose solution in 0.1 M PBS pH 7.4 for cryoprotection until it sank to the bottom of the jar.

The brains were cut in the standard frontal plane of the ferret atlas (Radtke-Schuller 2018) in 50 μm sections on a freezing microtome. Six separate consecutive sets of serial sections were collected in 0.1 M PB. Every third section (150 µm distance) was used to analyze the tracer labeling. At least one set of serial sections (every 300 μm) was counterstained with cresyl violet or neutral red. In some cases, an adjacent set was selected to visualize cytochrome oxidase activity.

### Tissue processing

CTB was visualized with immunohistochemistry reactions using a primary antibody goat anti-CTB, (dilution 1:15,000, List Biological Cat# 104 RRID: AB_2313636), a biotinylated secondary antibody [rabbit-anti-goat (CTB), dilution 1:200; Vector Laboratories Cat# BA-5000 RRID: AB_2336126], and 3,3′-diamino-benzidine (DAB; Sigma-Aldrich) as the chromogen (for details, see Bizley et al. 2015). FE and FR sections were mounted unstained and coverslipped with Vectashield for the examination of the fluorescent label (1 every 300 µm) and adjacent sections were counterstained with cresyl violet, neutral red, or cytochrome oxidase. WGA-HRP was visualized in one series of sections according to the standard tetramethylbenzidine technique and counterstained with neutral red (Mesulam 1978). One or two other series were stained with tungstate-stabilized tetramethylbenzidine which results in a reaction product that can be visualized in bright field and polarized light, and counterstained with cresyl violet (Llewellyn-Smith et al. 1993).

### Histological analysis

The size of the injection sites and the involvement of the cortical layers were analyzed after tissue processing. The effective volume of tracer uptake is difficult to determine, especially for fluorescent tracers. When DAB was used as chromogen, the zone of most intense reaction product visible in bright field was defined as the injection site. For the fluorescent tracers, the injection site sizes were estimated from the zone of brightest fluorescence emission (as observed under low light excitation). The injection sites estimated from the sections were assigned to the corresponding locations in appropriate atlas plates and used for an atlas based surface reconstruction in CorelDraw (Corel Corporation).

**Fig. 2:**
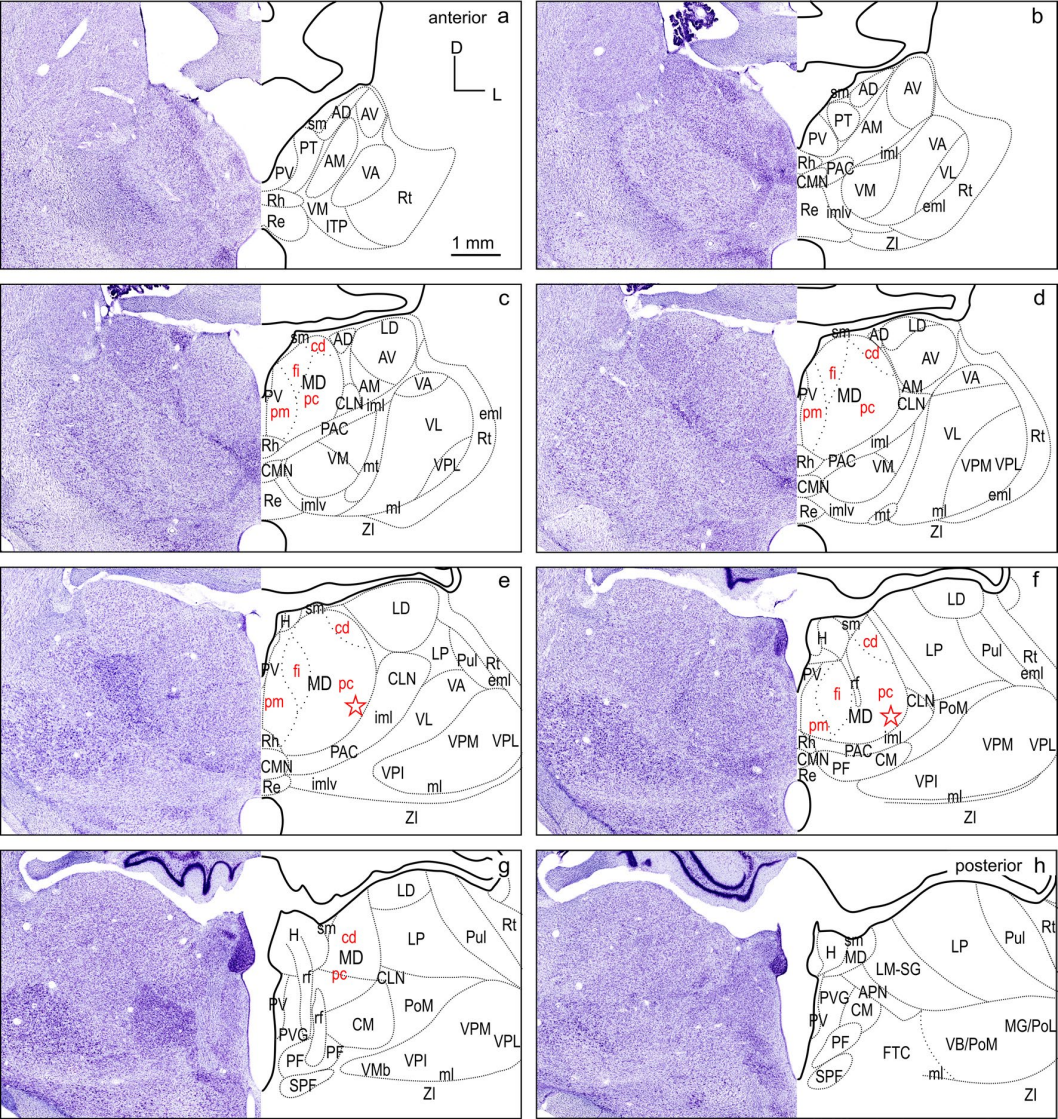
Thalamus of the ferret in frontal cell-stained sections. The panel represents the thalamic region at eight equidistant levels a-h (600 µm apart) modified from the ferret atlas (Radtke-Schuller 2018) plates 20–26 (cell stain). The black delineations of the thalamic nuclei on the right relate to the Nissl stained histology image on the left (mirrored). Subdivisions of MD indicated in red are based on their characteristic myelin pattern seen in Fig. 3. *AD* anterodorsal thalamic nucleus, *AM* anteromedial thalamic nucleus, *APN* anterior pretectal nucleus, *AV* anteroventral thalamic nucleus, *cd* pars caudodorsalis of MD, *CLN* centrolateral thalamic nucleus, *CM* centromedian thalamic nucleus, *CMN* central medial thalamic nucleus, *eml* external medullary lamina, *fi* pars fibrosa of MD, *FTC* central tegmental field, *H* habenula, *iml* internal medullary lamina, *imlv* internal medullary lamina ventral part, *ITP* nucleus of the inferior thalamic peduncle, *LD* laterodorsal thalamic nucleus, *LM-SG *lateralis medialis-suprageniculate complex, *LP* lateral posterior thalamic nucleus, *MD* mediodorsal thalamic nucleus, *MG/PoL* medial geniculate/posterior thalamic complex lateral region, *ml* medial lemniscus, *mt* mammillothalamic tract, *PAC* paracentral nucleus, *pc* pars parvicellularis of MD, *PF* parafascicular thalamic nucleus, *pm* pars paramediana of MD, *PoM* posterior thalamic complex medial region, *PT* paratenial thalamic nucleus, *Pul* pulvinar, *PV* paraventricular thalamic nucleus, *PVG* periventricular gray, *Re* reuniens thalamic nucleus, *rf* fasciculus retroflexus, *Rh* rhomboid thalamic nucleus, *Rt* reticular thalamic nucleus, *sm* stria medullaris of the thalamus, *SPF* subparafascicular thalamic nucleus, *VA* ventral anterior thalamic nucleus, *VB/PoM* ventrobasal complex/posterior thalamic complex medial region, *VL* ventrolateral thalamic nucleus, *VM* ventromedial thalamic nucleus, *VMb* basal ventral medial nucleus, *VPI* ventral posterior inferior nucleus, *VPL* ventral posterior lateral nucleus, *VPM* ventral posterior medial nucleus, *ZI* zona incerta. Star: presumed location of pars paralamellaris/multiformis in MD

**Fig. 3:**
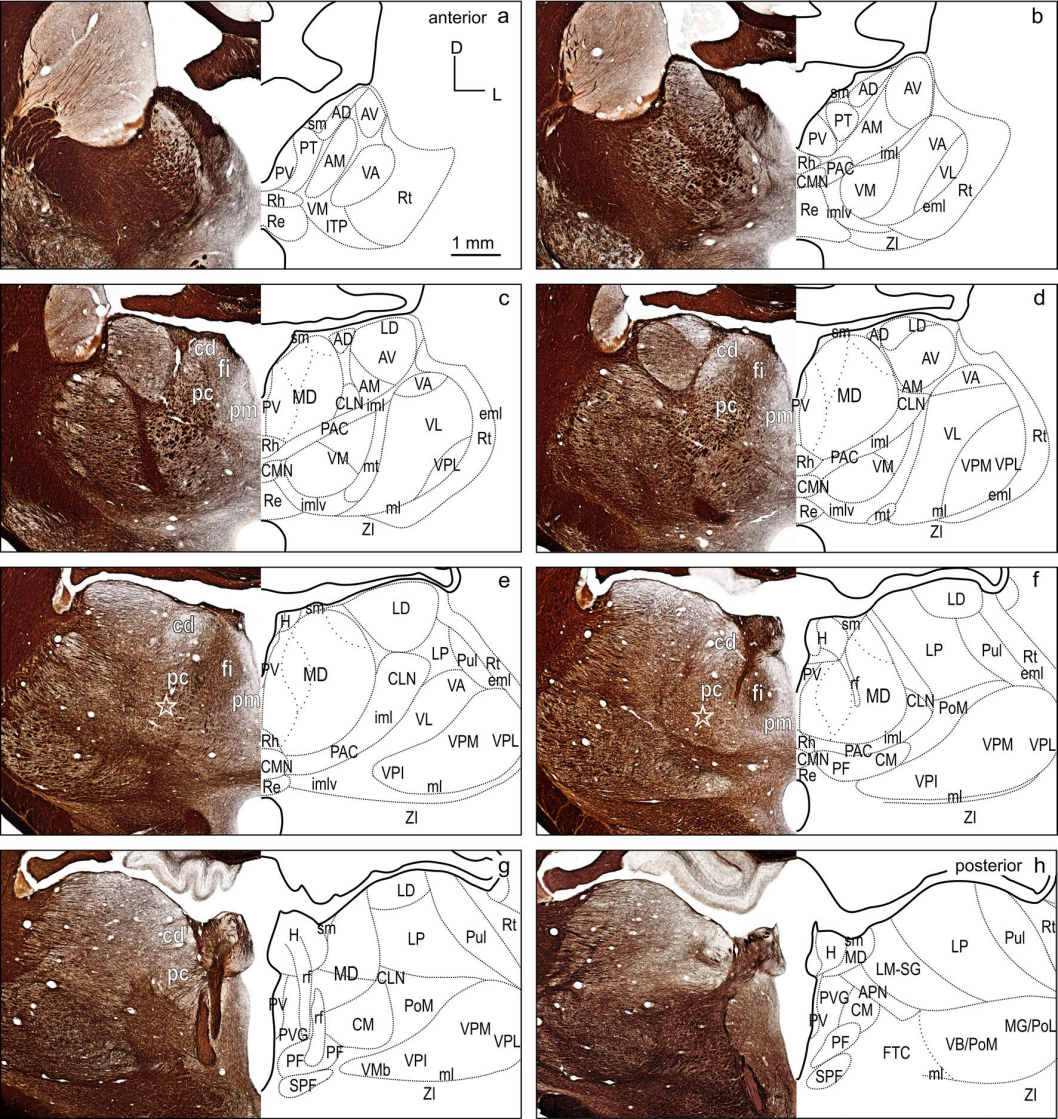
Thalamus of the ferret in frontal myelin stained sections. The panel represents the thalamic region at eight equidistant levels a–h (600 µm apart) modified from the ferret atlas plates 20–26 (myelin stain). The mirrored black delineations of the thalamic nuclei on the right are identical to those in Fig. 2. Subdivisions of MD based on their characteristic myelin patterns are marked in white in the histology image. For abbreviations see Fig. 2

To visualize the tracer label, the sections were scanned and digitized either in fluorescence, polarized light and/or bright field with a VS120 S1 microscope [Olympus BX61VST, with software dotSlide (Olympus)]. The magnification was 10 × optical and the resolution was 641 nm/pixel in both dimensions in the digital images. These images were imported into CorelDraw (Corel Corporation) and labeled cells were plotted in another plane to determine the location of labeled cells. To allow for a direct comparison of the labeling pattern in thalamus resulting from different FC injections, the labeled cells were registered on a common template. The template is based on cutouts of the thalamus region compiled from atlas plates 20–27 (Radtke-Schuller 2018), as shown in Figs. 2 and 3. It consists of eight subpanels spaced 600 µm and spans the anterior–posterior extent of thalamus from its rostral tip to the end of MD. The outlines of thalamic nuclei on the right are a mirror picture of the Nissl stained histology image on the left in Fig. 2. Subdivisions of MD are indicated based on their characteristic myelin pattern (myelin stain (Gallyas 1979)) seen in Fig. 3 following the definition by Ray and Price in the macaque monkey (Ray and Price 1993). For each experiment, the labeled cells of the sections best corresponding to the respective sub-panels were plotted as objects at the appropriate locations on the standardized template in CorelDraw.

The injection sites within FC were judged according to their coverage of dPFC and PMC. Two injections (one smaller and one larger injection) restricted to either dPFC or PMC were chosen for comparative analysis between fields. As the polar dPFC region might be a subdivision of PFC by itself, the two most rostral injections were treated as a separate group (dPFCpol) in the analysis. For the quantitative data collection, the number of labeled cells within all subdivisions in the template was evaluated by counting the number of objects using CorelDraw. This was done for each of the six representative cases, i.e., for two injection cases each in dPFC pol, dPFC, and PMC corresponding to the summary figure (Fig. 12) for these fields. Cases with injection sites involving more than one cortical field, or, with limited injection sites that did not span at least layers III, VI were excluded from quantitative analysis.

## Results

### Anatomical definition of ferret FC areas

The macroscopic and microscopic anatomy of the ferret’s dorsal FC subdivisions is depicted in top and side view of the ferret brain in Fig. 1a, b, and at two representative anterior–posterior levels in Fig. 1c, d. The nomenclature used is adapted from the ferret brain atlas (Radtke-Schuller 2018), which is based on the commonly used nomenclature of the dog FC proposed by Kreiner (1961).

In order to be compatible with previous nomenclature for the carnivore brain (for review, see Fuster 2015), the proreal gyrus (PRG) was introduced in the ferret atlas separately, instead of merging this dorsal rostral part of the ferret’s FC with the orbital gyrus (OBG) (Nigel et al. 1998; Kroenke et al. 2014). The PRG constitutes the anterior frontal lobe and mainly corresponds to the dPFC (Radtke-Schuller 2018). Ventrolaterally, it is roughly delimited by the proreal fissure (prof) adjacent to the OBG, medially, it borders on the medial FC (MFC). The posterior frontal lobe comprises the PMC in anterior sigmoid gyrus (ASG) and the motor cortex (M1 and MC) in posterior sigmoid gyrus (PSG), separated by the cruciate sulcus (crs) between the two gyri.

In this study, the term ‘dorsal FC’ refers to the dorsal cortical fields of the PRG and ASG of the frontal lobe.

The border between dPFC in PRG and PMC in ASG is not clearly visible as a sulcus, but can be roughly determined at the microscopic level, in that, the unstructured appearance of the proreal cortex (Fig. 1c, upper inset) gradually turns into a more layered one in the ASG (Fig. 1d, insets). In the upper layer V (d, star) pyramidal cells are visible as a dark band contrasting with a pale lower layer V. The size of the pyramidal cells within this band increases towards the PSG, where it reaches a maximum (‘giant pyramidal cells’) in M1 (not shown). The orbital cortex joins the dPFC lateroventrally on the OBG. Its layered appearance is mainly due to its distinct layer III (star in Fig. 1c, lower inset).

### Anatomical definition of ferret thalamic subdivisions

The thalamus nomenclature used in this study is mainly based on the terminology of Jones and Nieuwenhuys (Jones 1985; Nieuwenhuys et al. 2008), and largely in accord with the earlier work of Herbert (Herbert 1963) in the ferret. Subdivisions in MD were adapted from Ray and Price (Ray and Price 1993). The anatomical classification of the thalamus into groups of nuclei, according to their location (Table 1) was used to facilitate the synopsis of thalamic input sources to the different cortical areas (Figs. 2 and 3).

**Table 1:** Percentages of labeled thalamic cells projecting to the three differentiated FC fields dPFCpol, dPFC, and PMC

Nuclear group	Percentage of labeled cells in nuclear group referred to total number of labeled cells			Subdivision	Percentage of labeled cells in subdivisions referred to total number of labeled cells					
	dPFCpol	dPFC	PMC		dPFCpol	*N*	dPFC	*N*	PMC	*N*
	*N* = 965	*N* = 1805	*N* = 2535							
ANG	0	1.6	0.5	AD	0	0	0	28	0.04	13
				AM	0		1.44		0.43	
				AV	0		0.11		0.04	
MD	75.9	63.9	60.7	MDpm	6.42	732	1.39	1153	0.24	1540
				MDfi	32.02		9.03		0.32	
				MDpc	36.79		42.55		58.82	
				MDcd	0.62		10.91		1.38	
MiNG	5.3	3.1	0.9	PV	0	51	0.17	56	0.04	22
				PT	3.73		0.33		0.08	
				Rh	1.55		2.27		0.59	
				Re	0		0.33		0.16	
ING	16.1	13.3	10	PAC	6.22	155	3.38	240	4.38	254
				PAC/imlv	5.8		3.32		2.01	
				CLN	0		3.27		1.18	
				CMN	4.04		3.32		0.59	
				CM	0		0		1.62	
				PF	0		0		0.24	
VNG	2.4	7.6	22.2	VM	1.97	23	2.49	137	0.47	562
				VL	0		0		18.3	
				VPI	0		0		0	
				VPM	0		0		0.04	
				VPL	0		0		0.04	
				VA	0.41		5.1		3.31	
LNG	0.4	10	4.6	LD	0	4	2.77	181	0.08	117
				LM-SG	0.41		4.49		3.75	
				LP	0		2.77		0.59	
				Pul	0		0		0.2	

Percentages are compiled for thalamic nuclear groups in the left portion of the table. The second-to-fourth columns are referred to the total number of labeled cells resulting from the cortical field-specific injections, respectively (*N* indicated in the third row). The subdivisions of the nuclear groups are listed in the fifth column. The percentages of labeled cells in the subdivisions are referred to the total number of labeled cells given for the three differentiated frontal cortical fields in the sixth, eighth, and tenth columns. The adjacent column to the right indicates the total number of labeled cells in each nuclear group.

#### Subdivisions of MD

The primary subdivisions of the MD thalamus are the pars parvicellularis (MDpc), containing small cells, and the pars magnocellularis (MDmc), containing large cells. In primates, the MDpc and MDmc are clearly cytoarchitectonically distinguishable. In carnivores, including the ferret, these subdivisions are present, although less discernable in cell stain (cat and dog Akert 1964; dog Kosmal and Dabrowska 1980; Duque and McCormick 2010; Herbert 1963). Figures 2 and 3 show cell and myelin staining from adjacent sections through the ferret thalamus.

The myelin stain (Fig. 3) reveals that the ferret MD can be readily identified and subdivided in a manner consistent with subdivisions defined in the macaque monkey (Ray and Price 1993). The medial part of MD, MDmc, corresponds to a poorly myelinated area medially (pars paramediana; MDpm) and a laterally adjacent dense plexus of myelinated fibers (pars fibrosa; MDfi) (Fig. 3c–f, red labels). The large lateral MDpc is characterized by coarse fiber fascicles crossing from ventrolateral to dorsomedial. A further small poorly myelinated subdivision (pars caudodorsalis; MDcd) is discernable at the dorsolateral edge of MD anteriorly (Fig. 3c–e), which continues caudally more dorsomedially (Fig. 3f, g).

In some studies, a lateral MD part, that is not sharply contrasted in the myelin stain, is further distinguished along the lateral MD border, parallel to the fibers of the internal medullary lamina (iml) as pars paralamellaris (cat Rinvik 1968; cat, dog and monkey Akert 1964) or multiformis in macaque monkey (Olszewski 1952). In the ferret, pars paralamellaris presumably corresponds mainly to the lateral MDpc part, indicated by the stars in (Figs.2e, f and 3e, f).

### Localization of injection sites in dorsal FC

**Fig. 4:**
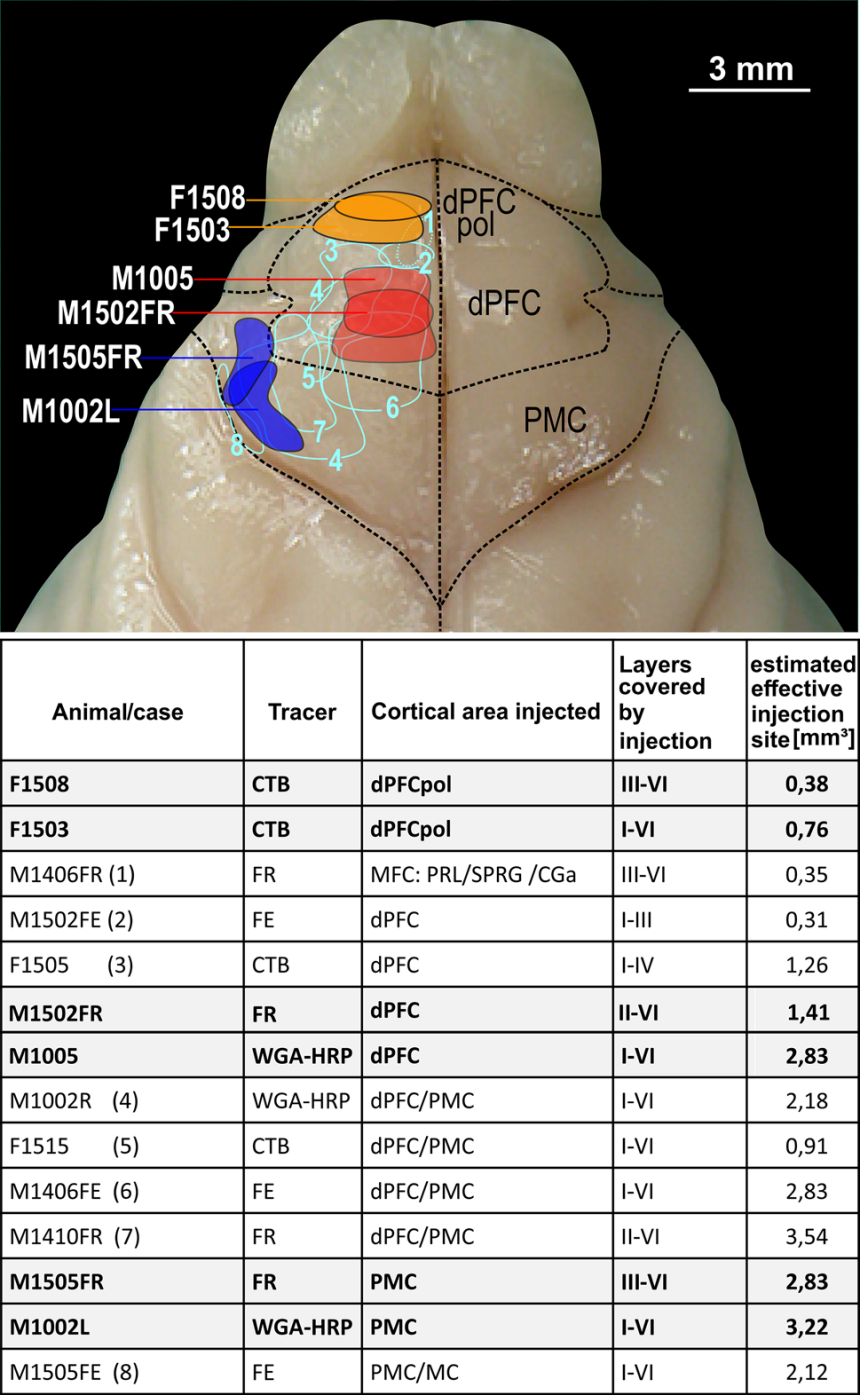
Overview of tracer injections into the frontal cortex. Upper part: injection sites are illustrated as areas projected and outlined on the left hemisphere of the ferret atlas brain within cortical field boundaries. The filled areas indicated by white animal numbers on the left highlight the experiments chosen for comparative analysis between fields (color code: orange dPFCpol, red dPFC, and blue PMC). All other experiments are demarcated by numbered contours. Lower part: in the table, all tracer injections made in this study (*N* = 14) are compiled giving details on tracer, cortical injection area, injection depth (cortical layers involved), and volume of estimated effective injection site. The cases chosen for comparative analysis between fields are highlighted and the numbers in parenthesis behind the animal number refer to the numbered contours of the cases in the upper figure part. *CGa* anterior cingulate gyrus, *dPFC* dorsal prefrontal cortex, *dPFCpol* polar region of dPFC, *MC* motor cortex, *MFC* medial frontal cortex, *PMC* premotor cortex, *PRL* prelimbic cortex, *SPRG* subproreal gyrus

To identify subdivisions within the dorsal FC on the basis of distinctive thalamic input patterns, different retrograde tracers were injected into various locations within the dorsal FC (14 injections in a total of ten ferrets). All injection sites were reconstructed from the histological material in reference to the atlas plates. Injection locations were delineated in the FC fields in a top view of the atlas brain shown in Fig. 4 (upper panel). The detailed injection parameters are summarized in the table in Fig. 4 (lower panel).

**Fig. 5:**
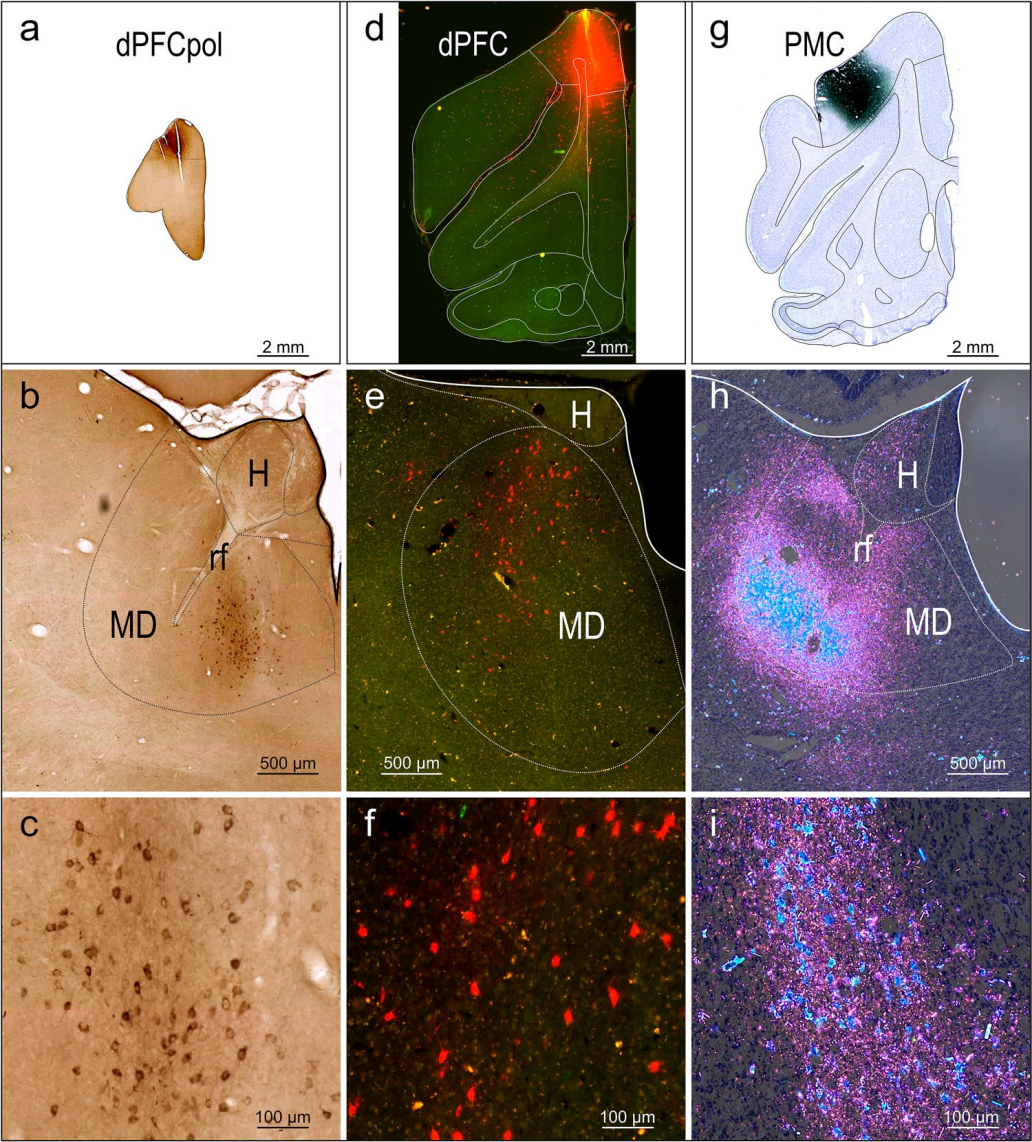
Original examples of tracer injections into FC areas and resulting label in MD. Left column: **a** injection site center of CTB tracer in dPFCpol in a frontal section (case F1503, Fig. 4). **b** Characteristic location of retrograde labeled cells in MDfi resulting from the tracer injection shown in a frontal section through the thalamus. With DAB used as chromogen, tracts of myelinated fibers stand out unstained against different shades of brown of the surrounding tissue and can be used for orientation within the section as ‘counterstain’. **c** Enlargement of the labelled MDfi cells. Middle column: **d** injection site center of FR tracer in dPFC (case M1502FR, Fig. 4) in a frontal section. The red fluorescence is contrasted by the green auto-fluorescence of the tissue and facilitates the localization of the FR labelled cells. **e** The cut out through the thalamus shows the red fluorescing labelled cells in dorsal MDpc. **f** The labelled MDpc cells at higher magnification. Right column: **g** injection site center of WGA-HRP tracer in PMC (case M1002L, Fig. 4) in a frontal section. The WGA-HRP was reacted with tungstate-stabilized tetramethylbenzidine which results in a reaction product that can be visualized in brightfield (**h**) and polarized light (**i**, **j**). Sections were counterstained with cresyl violet. The reaction product of retrograde labelled cells appears blue; anterograde label in pink. **k** Cut out of a frontal section through the thalamus showing the characteristic location of labeled cells in lateroventral MDpc. **l** Enlargement of labelled MDpc cells (image from another section with lower density of labeled cells to better demonstrate the blue stained cells within pink labeled anterograde transport reaction product). *dPFC* dorsal prefrontal cortex, *dPFCpol* dPFC polar region, *PMC* premotor cortex, *H* habenula, *MD* mediodorsal thalamic nucleus, *rf* fasciculus retroflexus

Figure 5 shows original examples of different retrograde tracer injections into the dorsal FC and the resulting label in MD.

### Thalamic input pattern of dorsal FC fields

**Fig. 6:**
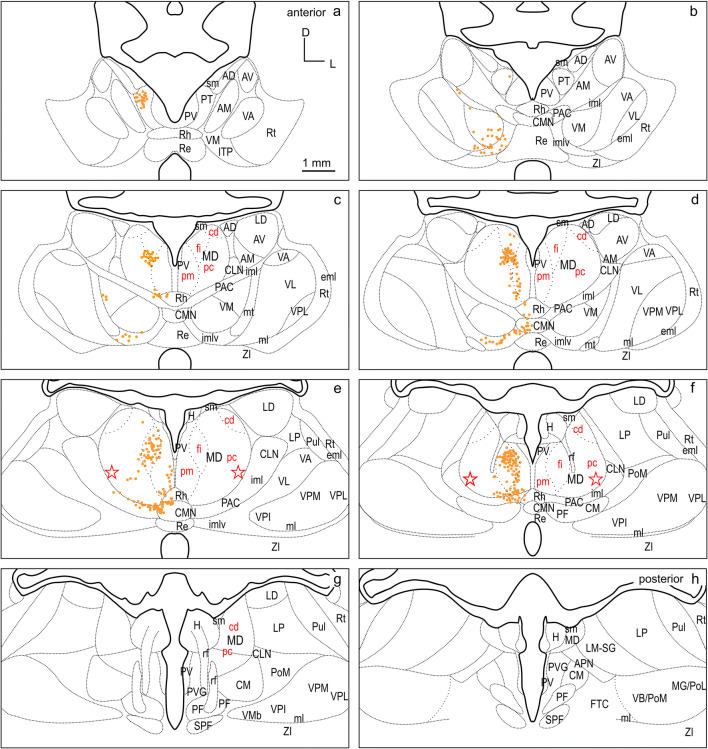
Retrograde labeled cells (orange dots) resulting from the smaller injection into dPFCpol (F1508 in Fig. 4) projected onto the thalamus template. For abbreviations see Fig. 2

**Fig. 7:**
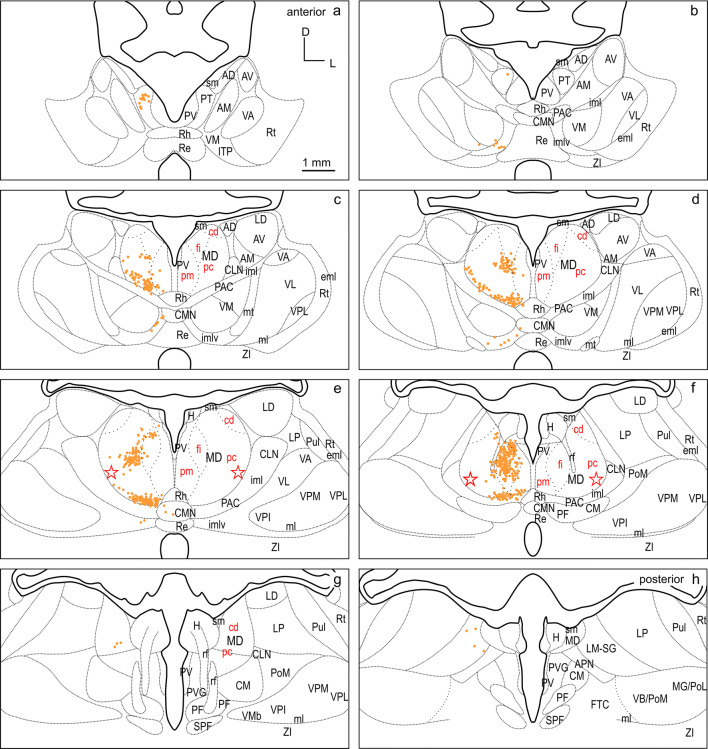
Retrograde labeled cells (orange dots) resulting from the larger injection into dPFCpol (F1503 in Fig. 4) projected onto the thalamus template. For abbreviations see Fig. 2

**Fig. 8:**
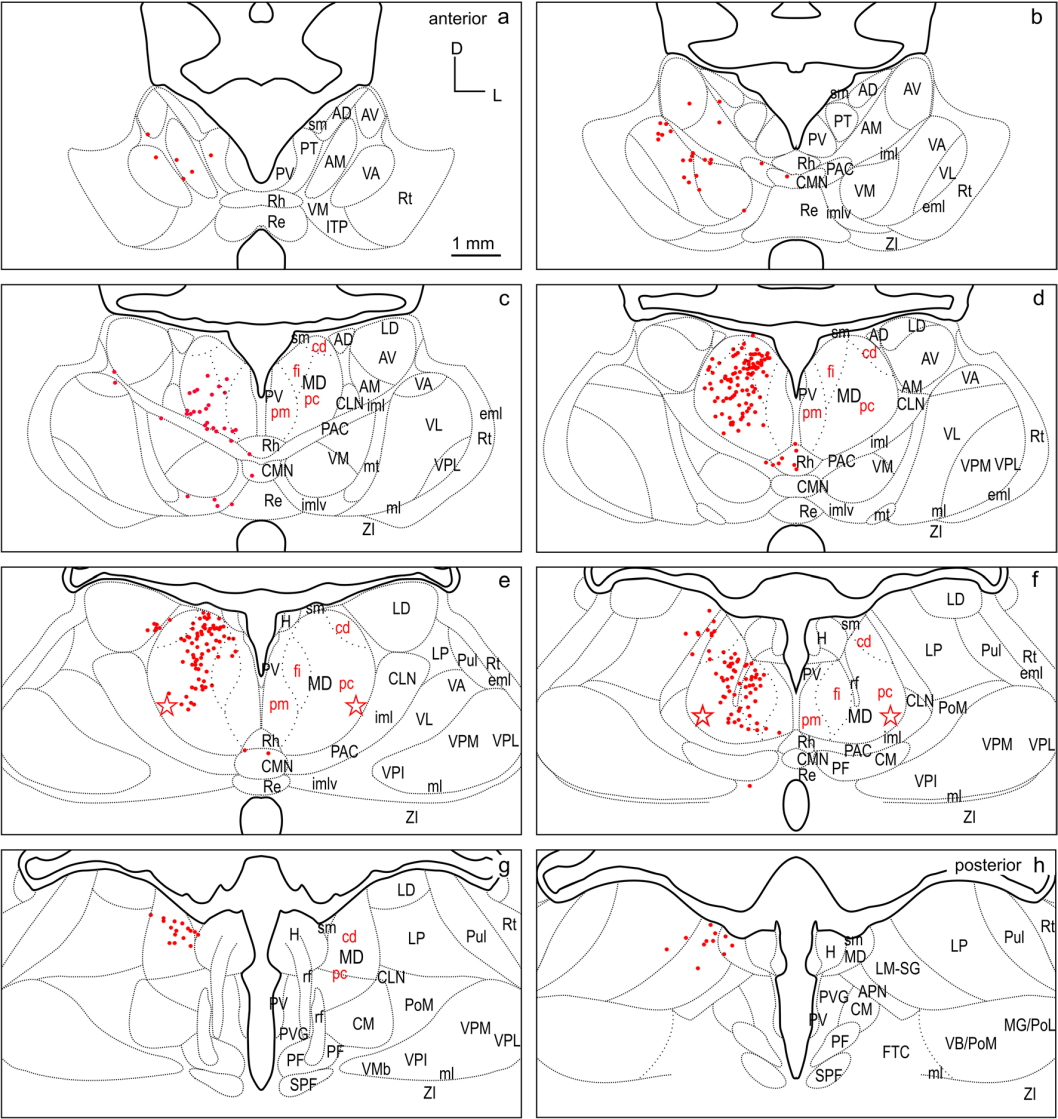
Retrograde labeled cells (red dots) resulting from the smaller injection into dPFC (M1502FR in Fig. 4) projected onto the thalamus template. For abbreviations see Fig. 2

**Fig. 9:**
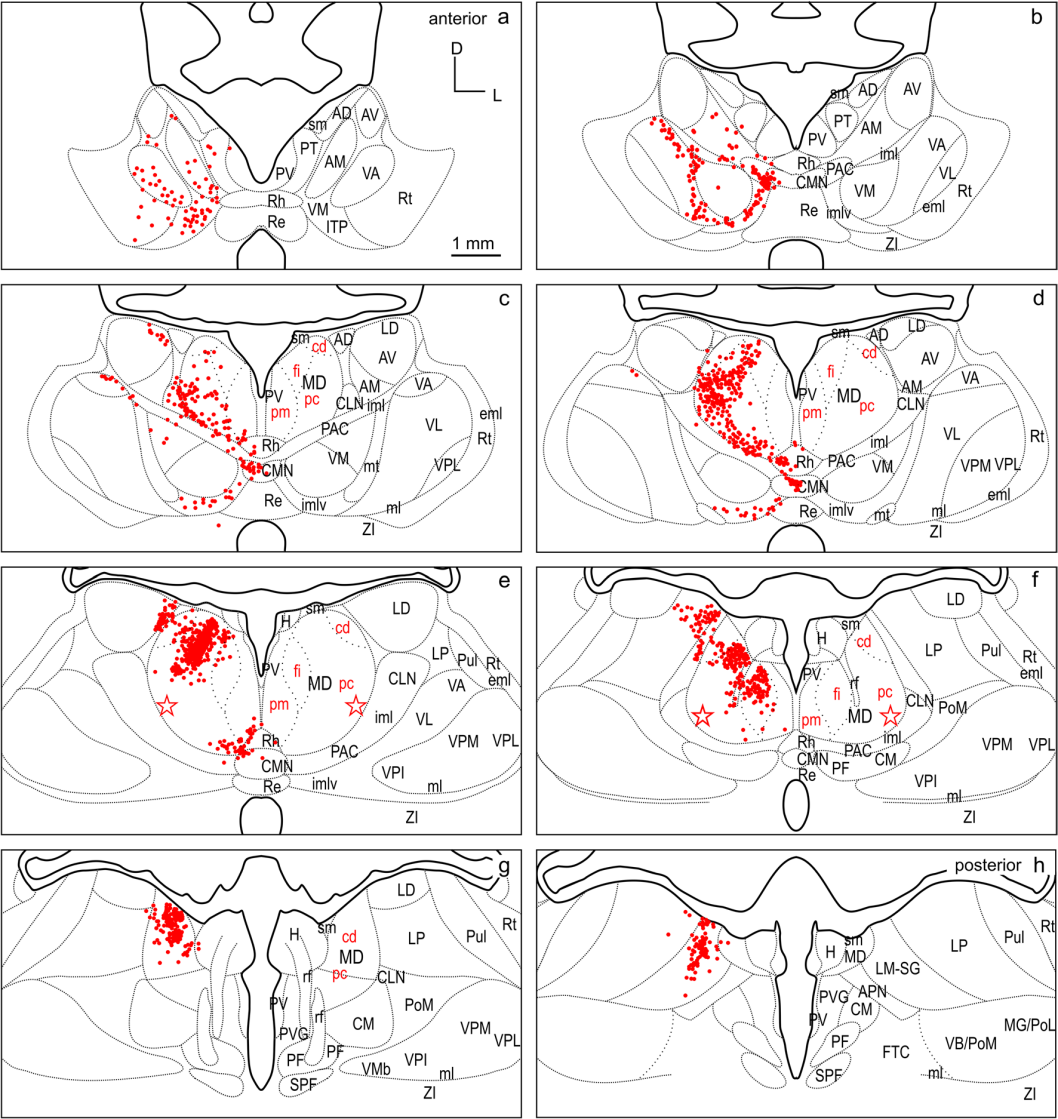
Retrograde labeled cells (red dots) resulting from the larger injection into dPFC (M1005 in Fig. 4) projected onto the thalamus template. For abbreviations see Fig. 2

**Fig. 10:**
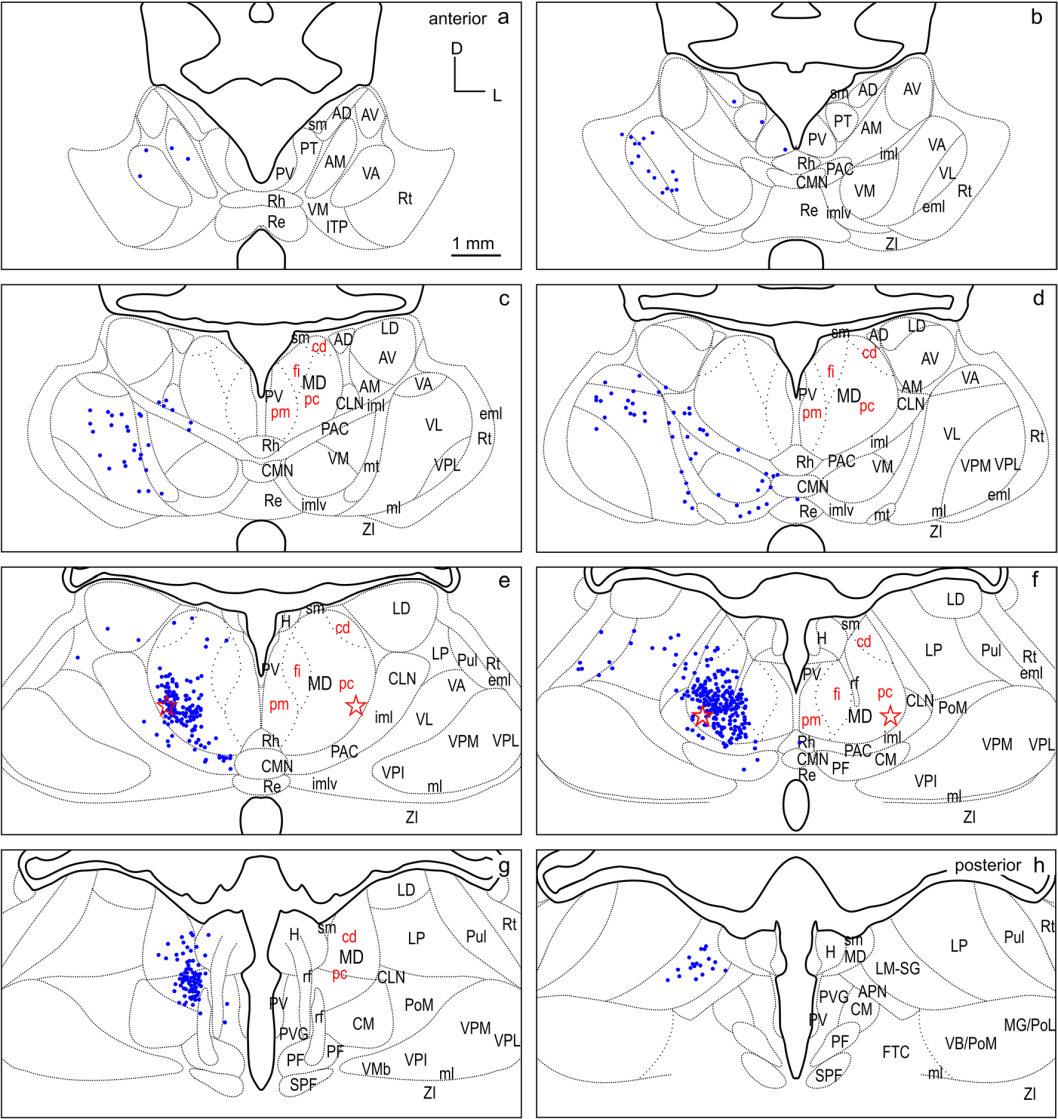
Retrograde labeled cells (blue dots) resulting from the smaller injection into PMC (F1505FR in Fig. 4) projected onto the thalamus template. For abbreviations see Fig. 2

**Fig. 11:**
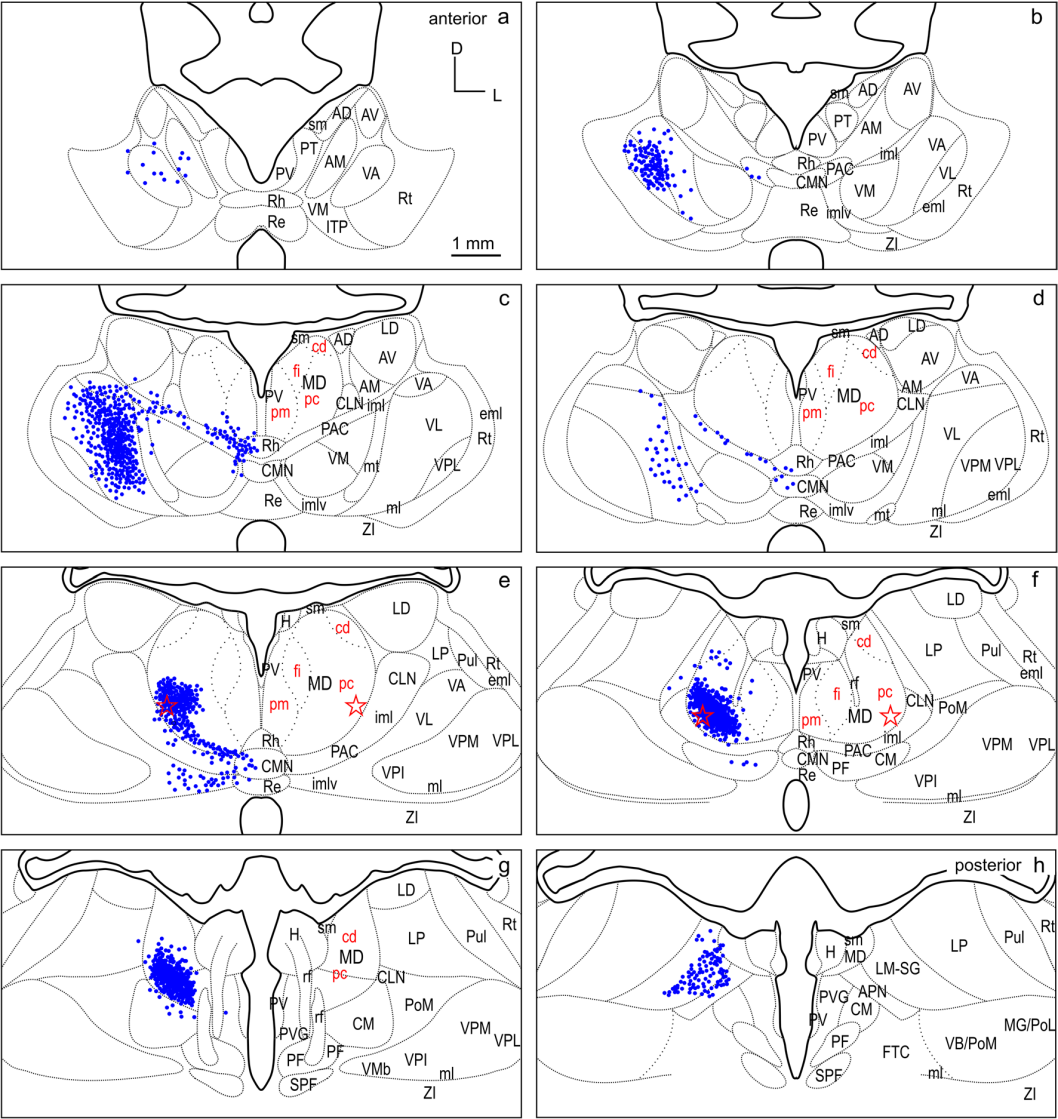
Retrograde labeled cells (blue dots) resulting from the larger injection into PMC (M1002L in Fig. 4) projected onto the thalamus template. For abbreviations see Fig. 2

**Fig. 12:**
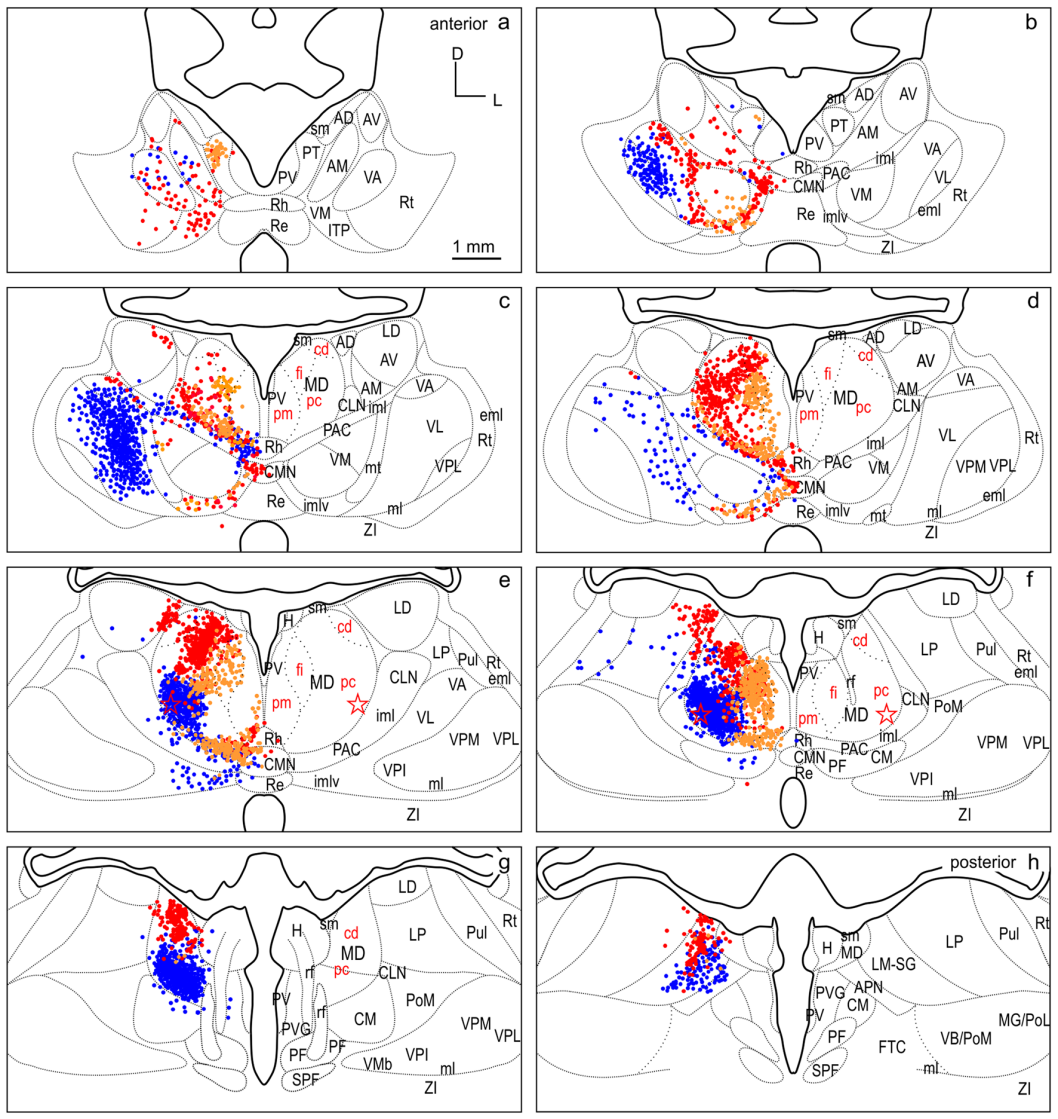
Summary of labeled cells of the six representative cases of injections into dPFCpol, dPFC, and PMC projected onto the thalamus template. Color coding is maintained as in Figs. 6, 7, 8, 9, 10, 11. Orange dPFCpol, red dPFC, blue PMC. For abbreviations see Fig. 2

Among the different cases, a larger and a smaller injection site confined to and characteristic of the three FC regions dPFCpol, dPFC, and PMC were selected for comparison of their corticothalamic projection patterns (Fig. 4, color-marked in orange, red, and blue, respectively). To compare labeling across animals, the retrograde label of each of the six representative cases was plotted on the same template of eight equidistant frontal sections through the thalamus (Figs. 6, 7, 8, 9, 10, 11, 12). The labeled cells resulting from injections into the three cortical regions of interest in dorsal FC are color-marked accordingly in orange for dPFCpol (Figs. 6, 7), in red for dPFC (Figs. 8, 9), and in blue for PMC (Figs. 10, 11). The labeling results, of the six representative cases with injections into all three areas, are summarized in Fig. 12 (same color-code) and Table 1. The boundaries of the thalamic nuclei of the template are based on the ferret atlas sections stained for cells (Nissl) and adjacent sections stained for myelinated fibers (Gallyas), as depicted in Figs. 2, 3, respectively.

### dPFCpol

CTB injections F1508 (smaller) and F1503 (larger) were confined to the polar part of dPFC (Fig. 4) and resulted in retrogradely labeled cells as depicted in Figs. 6, 7, respectively, and summarized in detail in Table 1.

The strongest input to dPFCpol originates from MD, with roughly three quarters of the total number (75.9%) of labeled thalamic cells (Table 1, left dPFCpol column). When the labeled cells are assigned to MD subdivisions, the overwhelming share comes from MDpc and MDfi, with few from MDpm and almost none from MDcd (Table 1, right dPFCpol column).

Beyond MD, the dPFCpol also receives considerable input (16.1%) from the intralaminar thalamic nuclear group (ING), from mainly the paracentral nucleus (PAC) and the part of PAC within ventral internal medullary lamina (PAC/imlv), and from central medial thalamic nucleus (CMN). The input from the midline nuclear group of thalamus (MiNG) is moderate (5.3%) and stems from the paratenial thalamic nucleus (PT) and rhomboid thalamic nucleus (Rh). A small projection (2.4%) from the ventral nuclear group of thalamus (VNG) targets the dPFCpol with afferents mainly from ventromedial thalamic nucleus (VM) and some from ventral anterior thalamic nucleus (VA). A very few cells of the lateralis medialis–suprageniculate nucleus (LM-SG), that belong to the lateral nuclear group (LNG), were also found to project to dPFCpol.

The pattern of labeled cells is very similar in both cases, and the minor differences between the two cases are probably due to the slight difference in rostro-caudal location and injection site size. The labeled cells of the larger, more caudal injection (F1503) lie slightly more laterally in MD, which is in accord with a general tendency across fields that more caudal FC locations receive input from more lateral portions of MD.

### dPFC

The distribution of labeled cells resulting from injections M1502FR (Fluororuby, smaller) and M1005 (WGA-HRP injection, larger) into the dPFC region (Fig. 4) is shown in Figs. 8, 9, respectively, and summarized in detail in Table 1.

Almost two-thirds (63.9%) of the total number of thalamic cells projecting to the two dPFC injection sites derive from MD (Table 1, left dPFC column). Considering the inputs from MD subdivisions alone, the strongest source to dPFC is MDpc, followed by MDcd and MDfi, and weakest from MDpm (Table 1, right dPFC column).

Thalamic inputs to the dPFC from outside the MD include afferent projections from the ING (amounting to 13.3% of the total thalamic inputs) which arise equally from PAC, PAC/imlv, centrolateral thalamic nucleus (CLN) and CMN. These inputs were more prominent following the larger injection. A significant input to dPFC arises in the LNG, accounting for 10% of the total thalamic projection neurons. Labeled cells were observed in three nuclei: LM-SG, laterodorsal thalamic nucleus (LD), and lateral posterior thalamic nucleus (LP). The dPFC also receives input from the VNG, which in total accounts for 7.6% of labeled thalamic cells. Within the VNG, labeled cells were observed in VA and VM with roughly twice as many observed in VA as in VM.

A smaller projection originates from the MiNG to dPFC and accounts for 3.1% of the total number of labeled thalamic cells. Cells in the MiNG originated in Rh and to a lesser extent in PT, reuniens thalamic nucleus (Re), and paraventricular thalamic nucleus (PV). A very weak projection (1.6%) was observed from the anterior nuclear group of thalamus (ANG) to dPFC, originating mainly in the anterior medial nucleus (AM). In addition to the inputs of the thalamic nuclear groups already described, a few labeled cells were observed in the lateral reticular nucleus of thalamus (Rt), the nucleus of the habenula (H), and the Zona incerta (ZI) (0.1%, 0.1%, and 0.4%, respectively).

A consistent pattern of labeling was observed after both of the two injections, and the label from the smaller injection appears as a subset of that observed after the larger injection.

### PMC

The thalamic cells labeled from M1505FR (Fluororuby injection, smaller) and M1002L (WGA-HRP injection, larger) into PMC (Fig. 4) are compiled in (Figs. 10, 11), respectively, and summarized in detail in Table 1.

As in the case with dPFCpol and dPFC, the strongest input to PMC comes from MD with 60.7% of the total number of thalamic cells projecting to the PMC injection sites (Table 1, left PMC column). Considering the input from MD alone, the major share comes from the caudal ventrolateral part of MDpc, to a lesser extent from MDcd and almost negligible from MDfi and MDpm (Table 1, right PMC column).

The VNG sends the second greatest projection to PMC, providing almost one quarter of the thalamic input to PMC (22.2%). In contrast to dPFC and dPFCpol, the projection arises predominantly from the ventrolateral thalamic nucleus (VL), with almost sixfold more cells than the smaller contribution from VA. Projections of VM are sparse. The ventral posterior medial nucleus (VPM) and ventral posterior lateral nucleus (VPL) provided almost negligible projections.

One-tenth of the thalamic inputs to PMC (10.0%) originate from the ING. Within the ING, the largest input arises in PAC and PAC/imlv, with smaller projections arising in centromedian thalamic nucleus (CM), CLN, CMN, and parafascicular thalamic nucleus (PF) in decreasing order. Projecting cells from LNG to PMC comprise a moderate thalamic input (4.6%). Projecting nuclei are LM-SG, LP, pulvinar (Pul), and LD.

PMC has very weak input (0.9%) from the MiNG. This input originates in Rh and Re, with PV and PT showing negligible contribution.

PMC also receives very sparse input (0.5%) from the ANG. Within ANG, input arises mainly from AM, and very sparse from the anterodorsal thalamic nucleus (AD) and the anteroventral thalamic nucleus (AV).

A few cells of the anterior pretectal area (APN), Rt, H, and the periventricular grey (PVG) were found to project to PMC. These connections together amount to 1% of the thalamic input to PMC.

The overall distribution of label between the two cases was very similar. The two injections differed slightly in their locations, with M1505FR being situated anterior to M1002L (Fig. 4). In M1002L, the number of labeled cells within VL is higher than in the more anterior case M1505FR.

### Differential input contribution of thalamic nuclei to dorsal FC fields

To directly compare the thalamocortical innervation of the three identified dorsal FC areas, the labeling from each of the six cases was overlaid in a summary figure demonstrating the distinct topographic distribution of thalamic projection sources to the dorsal FC fields (Fig. 12). The thalamic origins of the thalamo-cortical projections of the three dorsal FC areas have distinct topographic patterns and show distinct differences as well as some overlapping features.

**Fig. 13:**
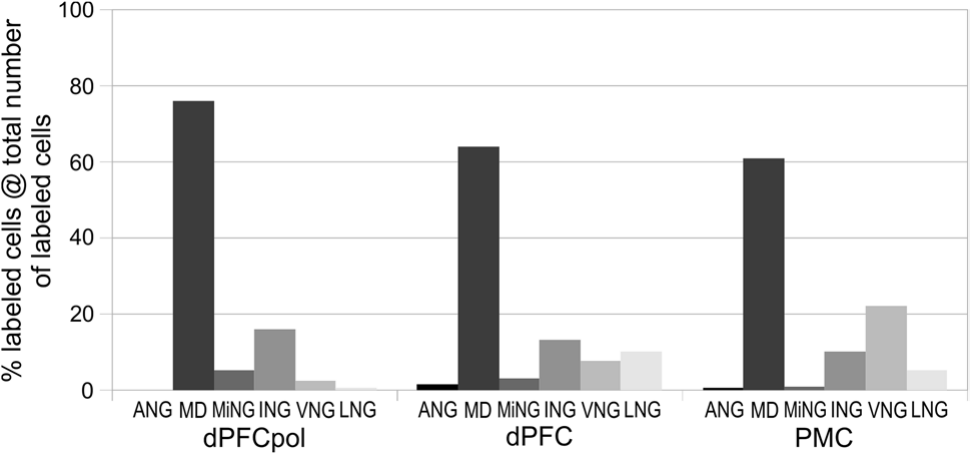
Contribution of projecting cells of thalamic nuclear groups to FC fields. The percentage of each group is referred to the total number of labeled cells resulting from the cortical field specific injections. The data for each cortical field comprise the two respective representative cases depicted in Figs. 6, 7, 8, 9, 10, 11, 12

**Fig. 14:**
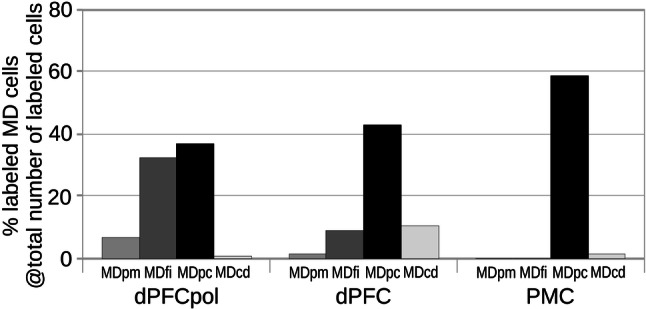
Contribution of MD subdivisions projecting to FC fields. The percentage of labeled cells in each MD subdivision is referred to the total number of labeled cells resulting from the cortical field specific injections. The data for each cortical field comprise the two respective representative cases depicted in Figs. 6, 7, 8, 9, 10, 11, 12

**Fig. 15:**
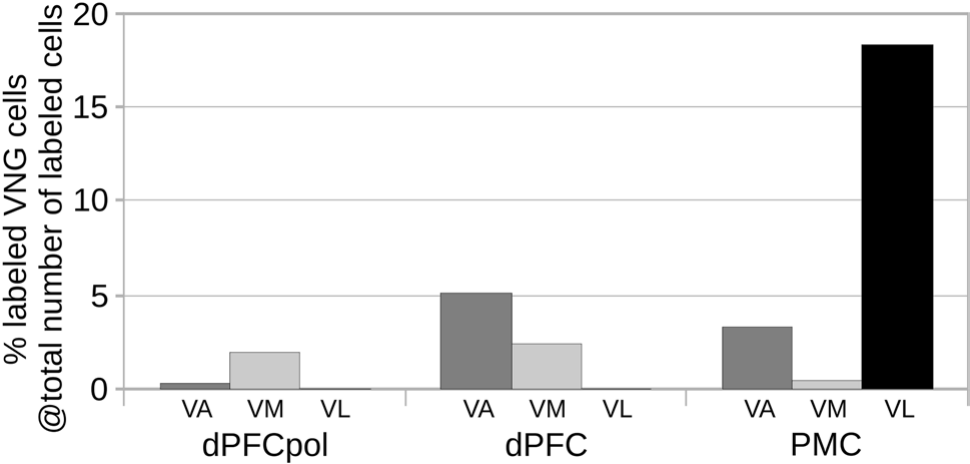
Contribution of VNG nuclei projecting to FC fields. The percentage of labeled cells of subdivisions VA, VL, and VM are referred to the total number of labeled cells resulting from the cortical field-specific injections. The data for each cortical field comprise the two respective representative cases depicted in Figs. 6, 7, 8, 9, 10, 11, 12

The quantitative input contributions of the different thalamic nuclei groups to each of the three cortical areas are presented in Fig. 13 and summarized in Table 1. The grey-shaded bar graphs represent the percentage shares of total number of labeled cells of the different thalamic nuclei groups for each cortical target area (the two representative cases for each area are combined). Input contributions from the MD and VNG nuclei are detailed similarly in Figs. 14 and 15.

### MD

In all three areas, the strongest thalamic input arises from MD, with a slight rostrocaudal gradient such that the polar region gets the strongest MD innervation (dPFCpol > dPFC > PMC), as shown in Fig. 13. All three cortical areas receive projections from all MD subdivisions, but with strongly diverging proportions (Fig. 14 and Table 1) and topographic differences (Fig. 12).

Among the MD subdivisions, MDpc provides the strongest projections to the three cortical areas and the main projection sources are topographically segregated. The medial and medioventral MDpc innervate dPFCpol, dorsolateral MDpc innervates dPFC, and the ventrolateral MDpc provides the dominant MD input to PMC. The ventrolateral portion of MDpc shows a more heterogenous projection pattern to the three cortical fields indicated by overlap of the color-coded cells in the summary Fig. 12 (see Figs. 6, 7, 8, 9, 10, 11 for a more detailed comparison). MDfi, a subdivision of MDmc, sends substantial projections to dPFCpol, much less to dPFC and extremely sparse to PMC. MDpm contributes moderately to the afferent projections of dPFCpol, sparsely to dPFC, and almost negligibly to afferents of PMC. MDcd contributes some input to dPFC, projects sparsely to PMC, and extremely sparse to dPFC pol.

### VNG

The most significant difference between the three cortical areas is their input from the VNG, which clearly differentiates the PMC from the other regions. PMC receives the greatest afferents from the VNG (PMC > dPFC > dPFCpol). In addition, the strong afferent projection from VL is a unique feature of PMC, as neither dPFCpol nor dPFC appear to receive any input from VL (Fig. 15 and Table 1). VA provides the main share of VNG projections to dPFC, about one-third less to PMC, whereas its projections to dPFCpol are sparse. VM projections dominate the VNG afferents to dPFCpol in comparison to the other VNG subdivisions. dPFC receives slightly more afferents from VM than dPFCpol, whereas PMC gets only little input from VM (Fig. 15). Very few scattered cells of the ventrobasal complex (VPM and VPL) project to PMC and no such projections were observed to dPFCpol and dPFC, and have, therefore, been omitted in Fig. 15.

### Other thalamic nuclei

Each of the three identified dFC areas receives distinct afferent projections from further thalamic nuclear groups. (Fig. 13 and Table 1). The ING sends substantial projections to all three areas, with a rostrocaudal gradient in terms of the overall contribution (dPFCpol > dPFC > PMC). However, the specific subdivisions projecting to each field differ. PAC and PAC/imlv substantially project to all three. CMN projects to dPFCpol and dPFC with equivalent strength, and to PMC rather more weakly. CLN projects substantially to dPFC and to PMC moderately. In contrast to dPFC and dPFCpol, only PMC receives input from CM and PF.

The input from the MiNG is smaller than that of the ING. Like MD, ING and MiNG projections show a decreasing gradient from rostral to caudal (dPFCpol > dPFC > PMC). Of the MiNG, PT almost exclusively projects to dPFCpol. Rh sends projections to all three regions. Re projections to dPFC and PMC are sparse; those of PV are very sparse.

The LNG contributes some input to dPFC, less to PMC and only very sparse to dPFCpol. The different LNG nuclei project with diverging proportions and topographic differences (see Fig. 12 and Table 1 for more detailed comparison). LM-SG projects to all three areas and has the largest share in projecting LNG neurons.

The ANG contributes the weakest input to all three areas, with a very small input to dPFC, almost negligible to PMC, and no input to dPFCpol.

Some projections to dPFC and PMC were observed from the most anterior Rt (0.4% and 0.2%, respectively). The nucleus of the lateral habenula (H) sends a few projections to dPFC and PMC (0.1% each). Sparse projections also come from paraventricular grey (PVG) to PMC (0.1%) and zona incerta (ZI) to dPFC (0.1%).

## Discussion

The present study, based on retrograde tracer injections in dorsal FC and quantitative analysis of the labeled thalamocortical projections, provides a detailed assessment of afferent connectivity of the thalamus with the fields of the ferret dorsal FC. In addition to the characteristic MD afferents, the dorsal FC fields receive specific input from multiple thalamic nuclei, establishing a unique connectivity pattern for each field and allowing for comparison with other species. The results confirm the cyto- and myeloarchitectonically based delineations of dPFC and PMC and provide additional evidence that the most anterior part of dPFC, dPFCpol, can be considered as a separate PFC subdivision as it exhibits a distinct thalamic input pattern. The thalamic connectivity of the ferret’s dorsal FC fields is consistent with the results of previous studies on other carnivores and non-human primates.

### MD parcellation

MD subdivisions preferentially and reciprocally connect with different FC areas and these separate thalamocortical circuits are part of functionally distinct networks. The designation of MD subdivisions is, therefore, crucial for the identification of FC fields. However, in the ferret, MD subdivisions are not easily defined by cytoarchitectural traits. Based on cell stained material, Herbert (Herbert 1963) and Duque (Duque and McCormick 2010) roughly subdivided MD into a medial and a lateral part, corresponding to the magnocellular and parvocellular MD, respectively, as described in other species. In comparison with cytoarchitecture, the myeloarchitectural characteristics within the ferret’s MD are clear and well developed, and allow for a comparison with the common myelin pattern-based definition of subdivisions in primates (Ray and Price 1993; Radtke-Schuller 2018). Nevertheless, the myelin stain also leaves some questions open, as subdivisions in the lateral and ventral MD are not clearly discernable. The lateral MDpc of Ray and Price (1993) includes the pars multiformis or pars paralamellaris of MD distinguished by others (e.g., dog, cat, monkey Akert 1964; rhesus monkey: Goldman-Rakic and Porrino 1985; Fang et al. 2006). In the ferret, the most likely region corresponding to pars paralamellaris is the postero-lateral MDpc (marked by a star in Fig. 3e, f and Figs. 6, 7, 8, 9, 10, 11, 12).

### Differential MD connectivity with dorsal FC fields

#### MD-dPFCpol

The polar region of dPFC receives a substantial input from MDmc (mainly MDfi) in addition to MDpc input of slightly larger size. MDmc has been found to be connected predominantly to orbital and medial areas in macaque monkey, with MDfi being reciprocally connected with central and lateral orbital FC (Brodmann Areas (BA) 11–13) and MDpm being reciprocally connecting with caudal and medial orbital FC (BA 13 and 14) (Ray and Price 1993; for review, see Mitchell and Chakraborty 2013). In addition, as in the ferret, MDmc together with MDpc also targets the frontal pole cortex (BA 10) in non-human primates (Burman et al. 2011a). The ferret’s dPFCpol is quite different from BA10 in non-human primates and Brodmann (1909) judged the polar cortex in carnivores (Kinkajou) in his comparative cytoarchitectural mapping study as belonging to BA 8. Further studies of the neuroanatomical cortical and subcortical connectivity of dPFCpol are key to understanding its role, as has been explored in recent connectional studies of BA 10 in non-human primates (Burman et al. 2011a, 2011b; Rosa et al. 2019). However, the presence of afferents from both MDmc and MDpc, the ‘limbic’ part and the ‘cognitive’ part of MD, respectively, suggest that ferret dPFCpol may play a role in comparing, integrating and rating internal and external signals and needs for decision making and selective attention. It is tempting to speculate that the polar part of dPFC in ferrets may, therefore, be involved in simple forms of “managing competing goals”, a key function of the frontopolar cortex, as proposed in humans (Mansouri et al. 2017). However, clearly more research on its neurophysiological activity during behavior is necessary to further evaluate the functional role of the ferret dPFCpol.

#### MD-dPFC

The ferret dPFC connectivity with MD is similar to that of other carnivores that have been studied. It matches projections described in the cat from a central sector of MD to the gyrus proreus, the dorsal PFC (e.g., Akert 1964; Markowitsch et al. 1978). Compared with the dog, the ferret organization pattern of MD projections to the dorsal FC fields also appears to be remarkably similar. The dog’s proreal gyrus, the dorsal PFC region, mainly receives projections from the intermediate–dorsal part of MD, which is believed to correspond to the parvocellular MD subdivision (Kosmal 1981a; Narkiewicz and Brutkowski 1967).

Of course, since there is no granular FC in the ferret, possible cortical cytoarchitectonic homologies with non-human primates are less clear (Wise, 2008). However, from a connectional perspective, the MDpc projections to dPFC in the ferret are highly comparable to similar projections of MDpc to the dorsolateral prefrontal cortex DLPFC in the monkey (Akert 1964; Goldman-Rakic and Porrino 1985; Ray and Price 1993; Erickson and Lewis 2004; Fang et al. 2006).

Ray and Price (1993) found that MD subdivisions in the monkey as defined cyto- and myeloarchitecturally and based on connectivity are also “…comparable to similar projections in the rat, to the extent that homologies can be drawn between the two species” (pages 27–28). Accordingly, if these cross-species parallels are correct, then MDpc in monkey, and in the ferret as discussed above, would be analogous to the ventrolateral MD segment in the rat. The corresponding projection targets are in the dorsolateral prefrontal or premotor areas in all three species, with BA 45, 46, 6, 8, 9 and, to some extent, BA12 in the monkey, dPFC and PMC in the ferret, and areas PrCm (medial precentral cortex), FPl (lateral frontal polar cortex), and DLO (dorsolateral orbital cortex) in the rat (Ray and Price, 1993).

#### MD-PMC

The ferret is also similar to the cat in sharing projections from lateral or paralamellar MD to the cortex posterior to PFC (Vedovato 1978; Moran and Reinoso-Suárez 1988) which corresponds to the premotor cortex (Area 6a-β (PMC) in the cat). It has been suggested by some that this area corresponds to the FEF (frontal eye field) in the monkey (Akert 1964; Markowitsch et al. 1978; Cavada and Reinoso-Suárez 1985), a highly controversial assumption that has not been confirmed (Schlag and Schlag-Rey 1970; Guitton et al. 1978).

As in the ferret, strong posterolateral MD input to premotor cortex is found in the dog, that the neighboring dPFC is lacking (Kosmal 1981b; Stanton et al. 1986; Sakai et al. 1993).

The MD sources of projections to PMC in the ferret are the most lateral and caudal parts of MDpc (marked by a star in Figs. 10, 11, 12e, f). These are likely to correspond to pars paralamellaris of Akert (1964) and MD multiformis in the monkey (Morel et al. 2005; Fang et al. 2006; Stepniewska et al. 2007), which are origins of MD projections to dorsal PMC and/or FEF.

However, our knowledge of the ferret FC is still incomplete and the motor cortex has not been fully mapped. The number of subdivisions of the ferret motor cortex is yet unknown and, therefore, all the presumed motor cortical areas of ASG are subsumed as ‘PMC’. Since the location of FEF in the ferret is presently unknown, conclusions about possible homologies with other species remain elusive at this stage.

### Differential VNG connectivity with dorsal FC fields

The VNG is traditionally thought to constitute the ‘motor’ thalamus. Otherwise, it has long been established that the thalamic motor nuclei are also part of basal ganglia circuits, such as the most prominent ‘dorsolateral prefrontal loop’ (Kandel et al. 2000; Purves 2004). These circuits are believed to play a key role in the functional regulation of ‘non-motor’ areas of the neocortex, including the prefrontal, associative, sensory, and limbic areas (Benagiano et al. 2018).

In carnivores (cat: Vedovato 1978; Katayama et al. 1986; Moran and Reinoso-Suárez 1988; dog: Kosmal 1981a; Kosmal 1986; Sakai et al. 1993) and non-human primates (e.g., Schell and Strick 1984; Matelli et al. 1989; Morel et al. 2005, Fang et al. 2006), VA and VM are involved in projections to all FC fields, but mainly to non-motor areas, whereas VL provides the major input to areas of motor cortex. The connectivity of VNG with the different dorsal FC fields in the ferret is consistent with these findings in other mammals and supports the distinction of the cortical fields, dPFC and PMC. All three dorsal FC areas of the ferret receive input from VNG with increasing projection strength from rostral to caudal and specific projection patterns from the VNG subdivisions. All three dorsal FC areas are targeted by VA and VM, but only PMC has considerable input from VL.

In the ferret, as also described in the dog, an afferent projection from VL in combination with strong posterolateral MD input is a unique feature of PMC that the neighboring dPFC is lacking (Kosmal 1981b; Stanton et al. 1986; Sakai et al. 1993). Furthermore, studies in rats, cats, dogs, and primates have shown that besides the classical motor nuclei, the anterior and posterior intralaminar nuclei together with the adjacent lateral MD portion, can also be considered to be part of motor thalamus, as they too receive cerebellar and/or basal ganglia input (Hintzen et al. 2018). In the ferret, these regions project to PMC.

### Differential ING, MiNG, LNG, and ANG connectivity with dorsal FC fields

ING and MiNG are higher order thalamic nuclei and are thought to play a role in awareness and to be involved in cognitive functions such as learning, memory processes, behavioral flexibility, and arousal regulation (Van Der Werf et al. 2002; Vertes and Hoover 2008; Cassel et al. 2013; Varela et al. 2014). These nuclei potentially modulate the degree of synchrony between different groups of cortical neurons according to behavioral demands (Saalmann 2014; Varela et al. 2014).

Based on remarkable similar connectivity patterns of various mammals (mainly rats, cats, and monkeys), Van der Werf et al. (2002) propose a classification of the midline and intralaminar nuclei (MiNG and ING, respectively) into four groups with different functions, which also applies to the ferret and sheds light on possible functions of the different fields.

According to this classification, all three dorsal FC fields of the ferret receive substantial input (dPFCpol > dPFC > PMC) from the *lateral group* that has cognitive functions and plays a role in ‘cognitive awareness’, enabling flexibility in the use of cognitive strategies. These inputs correspond to those from PAC and PAC/imlv, CMN, and also CLN in the ferret.

The dPFCpol additionally receives substantial input from the nuclei of the *dorsal group*, which have viscero-limbic functions and are critically involved in affective behaviors (Van Der Werf et al. 2002; Hoover and Vertes 2007; Kirouac 2015; Vertes et al. 2015). These inputs correspond to PT and medial MD in the ferret. The ferret’s dPFC and PMC receive only minor input from PT and even less from PV and the medial MD.

A few projections to PMC also come from PM and PF belonging to the *posterior group*, which is motor related and plays a role in modulation of motor responses (following awareness of salient stimuli).

Rh and Re, the nuclei of the *ventral group*, were proposed to play a role in polymodal sensory awareness (Van Der Werf et al. 2002) and also may contribute to learning, memory consolidation, and behavioral flexibility (Cassel et al. 2013). Rh/Re mainly target limbic cortical structures, particularly the hippocampus and medial PFC and participate in functions involving the interactions of the hippocampus formation and medial PFC. However, some efferents from Rh/Re were found to project to the ferret’s dorsal FC fields, with a greater share projecting to dPFC and PMC compared to dPFCpol.

LNG contributes a moderate projection mainly from LM-SG to dPFC and PMC, less from LP and LD. Like ING and MiNG, the LNG might contribute to the control of cortical information transmission (Saalmann 2014; Perry and Mitchell 2019).

From ANG, which has limbic-association functions, only a negligible projection to dorsal FC was observed in this study.

### Unlabeled ‘blank’ areas in thalamus after dorsal FC injections

We observed sparsely labeled thalamic ‘islands’ that showed only a few labeled cells after dorsal FC field injections. These ‘blank’ areas were found in MDpm, MDcd, and VM, thalamic nuclei known to project to FC. We speculate that these unlabeled regions mainly project to FC areas that were not injected or only marginally involved, i.e., the medial and orbital PFC and possibly also the dorsomedial PMC.

This view is supported by the studies of the connectivity of these thalamic subdivisions in different animal species. In the dog (Narkiewicz and Brutkowski 1967; Tanaka 1977; Kosmal and Dabrowska 1980), the medial, magnocellular MD is described as a source for projections to the orbital gyrus. In the ferret, the marginal input from MDpm to dPFC and PMC observed in this study (only dPFCpol received significant input from MDpm) is in line with the finding in macaques that MDpm projects to more caudal orbital regions that were not included in the injections. MDfi, on the other hand, is reciprocally connected with orbitofrontal cortex in macaque monkeys (Ray and Price 1993) and constitutes the main MDmc input to dPFCpol in the ferret.

MDcd input was insignificant besides the projections observed to dPFC in this study. As MDcd projects to anterior cingulate cortex in macaques (Ray and Price 1993), the reason for the projections from MDcd to dPFC might be some incidental spread of the dPFC injection sites (M1005 and M1502FR) into the neighboring dorsal anterior cingulate cortex of MFC.

VM projections in the cat target prefrontal cortex, including ventrolateral orbital cortex (Craig et al. 1982), premotor and rostral agranular insular cortices (Martínez-Moreno et al. 1987). In the dog, VM sends projections to the whole PFC including orbital and medial PFC, and PMC (Kosmal 1986). Significant label in orbital PFC after large tracer injections aimed at MD was also seen in the ferret, in cases that involved the medial and ventral MD and most probably VM (Duque and McCormick 2010).

## Summary and conclusions

This neuroanatomical study extends the previous results on thalamic projections to ferret dorsal FC (Duque and McCormick 2010). The three dorsal FC fields of the ferret are predominantly connected with MD and show distinct topographical projection patterns with different subdivisions of the nucleus, as suggested by the previous studies in different mammalian species. The more dorsolateral parvocellular division (MDpc) favors the dorsolateral and lateral cortex, and projects to dPFC in the ferret corresponding to DLPFC connectivity in non-human primates. The lateral (paralamellar/multiform) MD targets PMC and possibly the hitherto undefined FEF in the ferret and PMC and FEF in other carnivores and non-human primates. The polar region of dPFC receives a substantial input from MDmc (mainly from MDfi) in addition to MDpc input of slightly larger size. This MD projection pattern resembles that of the frontal pole cortex (BA 10) as described in non-human primates. Furthermore, the differential projection pattern of the ventral thalamic nuclei to the three dorsal FC fields with VL projecting only to PMC, as well as the differential projection patterns of thalamic intralaminar and midline nuclei, are all consistent with findings in other mammals and support the distinction of the cortical fields of dPFC and PMC in the ferret.

The observed remarkable similarities of the ferret’s dorsal FC with other species are based on an analysis of the thalamic input patterns. Additional studies exploring the connectome of the ferret’s dorsal FC fields, especially their cortico-cortical connectivity, will be necessary for a deeper understanding of ferret dorsal FC and possible homology with other species. Further behavioral and physiological data with precise anatomical localization will help to more fully comprehend the functional roles of the different dorsal FC fields in the ferret and their differential contributions to decision-making, attention, top-down cognitive control of sensory processing, and adaptive goal-directed behavior. The patterns of substantial projections from the thalamic nuclear groups, especially from MD and from VNG to the dorsal frontal areas, suggest some common functional properties, such as have been described and discussed in the primate and non-human primate PMC and dorsal prefrontal areas (e.g., Badre and D’Esposito 2009; Romo and de Lafuente 2013). Recent promising neurophysiological studies of ferret dorsal FC, in conjunction with renewed neuroanatomical and behavioral studies, suggest that the ferret may be well suited to becoming an ideal carnivore model for future research to elucidate and refine our understanding of frontal cortex structure and function.

## Abbreviations

ACG: Anterior composite gyrus
AD: Anterodorsal thalamic nucleus
AM: Anteromedial thalamic nucleus
ANG: Anterior nuclear group of thalamus
APN: Anterior pretectal nucleus
ASG: Anterior sigmoid gyrus
AV: Anteroventral thalamic nucleus
BA: Brodmann Area
CGa: Anterior cingulate gyrus
cd: Pars caudodorsalis of MD
CLN: Centrolateral thalamic nucleus
CM: Centromedian thalamic nucleus
CMN: Central medial thalamic nucleus
crs: Cruciate sulcus
CTB TRITC: Cholera toxin B subunit tetramethylrhodamine B isothiocyanate conjugate
DAB: 3, 3′-Diamino-benzidine
dPFC: Dorsal PFC
dPFCpol: DPFC polar region
eml: External medullary lamina
FC: Frontal cortex
fi: Pars fibrosa of MD
FE: Fluoro-Emerald
FEF: Frontal eye field
FR: Fluoro-Ruby
FTC: Central tegmental field
H: Habenula
iml: Internal medullary lamina
imlv: Internal medullary lamina ventral part
ING: Intralaminar thalamic nuclear group of thalamus
ITP: Nucleus of the inferior thalamic peduncle
LD: Laterodorsal thalamic nucleus
LM-SG: Lateralis medialis-suprageniculate complex
LNG: Lateral nuclear group of thalamus
LP: Lateral posterior thalamic nucleus
MC: Motor cortex
M1: Primary motor cortex
MD: Mediodorsal thalamic nucleus
MDcd: Pars caudodorsalis of MD
MDfi: Pars fibrosa of MD
MDmc: Pars magnocellularis of MD
MDpc: Pars parvicellularis of MD
MDpm: Pars paramediana of MD
MFC: Medial frontal cortex
MG/PoL: Medial geniculate/posterior thalamic complex lateral region
MiNG: Midline nuclear group of thalamus
ml: Medial lemniscus
mt: Mammillothalamic tract
OBG: Orbital gyrus
ofs: Olfactory sulcus
PAC: Paracentral nucleus
pc: Pars parvicellularis of MD
PF: Parafascicular thalamic nucleus
PFC: Prefrontal cortex
pm: Pars paramediana of MD
PMC: Premotor cortex
PoM: Posterior thalamic complex medial region
PRG: Proreal gyrus
PRL: Prelimbic cortex
prof: Proreal fissure
prs: Presylvian sulcus
PSG: Posterior sigmoid gyrus
PT: Paratenial thalamic nucleus
Pul: Pulvinar
PV: Paraventricular thalamic nucleus
PVG: Periventricular gray
Re: Reuniens thalamic nucleus
rf: Fasciculus retroflexus
rfa: Rhinal fissure anterior part
Rh: Rhomboid thalamic nucleus
Rt: Reticular thalamic nucleus
S1: Primary somatosensory cortex
sm: Stria medullaris of the thalamus
SPF: Subparafascicular thalamic nucleus
SPRG: Subproreal gyrus
VA: Ventral anterior thalamic nucleus
VB/PoM: Ventrobasal complex/posterior thalamic complex medial region
VL: Ventrolateral thalamic nucleus
VM: Ventromedial thalamic nucleus
VMb: Basal ventral medial nucleus
VNG: Ventral nuclear group of thalamus
VPI: Ventral posterior inferior nucleus
VPL: Ventral posterior lateral nucleus
VPM: Ventral posterior medial nucleus
WGA-HRP: Wheat germ agglutinated horseradish peroxidase
ZI: Zona incerta

## References

[CR1] Akert K, Warren JMAK (1964). Comparative anatomy of frontal cortex and thalamofrontal connections. The frontal granular cortex and behavior.

[CR2] Akert K, Hartmann-von Monakow K (1980). Relationships of precentral premotor and prefrontal cortex to the mediodorsal and intralaminar nuclei of the monkey thalamus. Acta Neurobiol Exp (Wars).

[CR3] Badre D, D’Esposito M (2009). Is the rostro-caudal axis of the frontal lobe hierarchical?. Nat Rev Neurosci.

[CR4] Benagiano V, Rizzi A, Lorusso L (2018). The functional anatomy of the cerebrocerebellar circuit: a review and new concepts. J Comp Neurol.

[CR5] Bimbard C, Demene C, Girard C (2018). Multi-scale mapping along the auditory hierarchy using high-resolution functional ultrasound in the awake ferret. Elife.

[CR6] Bizley JK, Bajo VM, Nodal FR, King AJ (2015). Cortico-cortical connectivity within ferret auditory cortex. J Comp Neurol.

[CR7] Brodmann K (1909). Vergleichende lokalisationslehre der großhirnrinde.

[CR8] Burman KJ, Reser DH, Richardson KE (2011). Subcortical projections to the frontal pole in the marmoset monkey. Eur J Neurosci.

[CR9] Burman KJ, Reser DH, Yu HH, Rosa MGP (2011). Cortical input to the frontal pole of the marmoset monkey. Cereb Cortex.

[CR10] Carlen M (2017). What constitutes the prefrontal cortex. Science.

[CR11] Cassel JC, Pereira de Vasconcelos A, Loureiro M (2013). The reuniens and rhomboid nuclei: neuroanatomy, electrophysiological characteristics and behavioral implications. Prog Neurobiol.

[CR12] Cavada C, Reinoso-Suárez F (1985). Topographical organization of the cortical afferent connections of the prefrontal cortex in the cat. J Comp Neurol.

[CR13] Craig AD, Wiegand SJ, Price JL (1982). The thalamo-cortical projection of the nucleus submedius in the cat. J Comp Neurol.

[CR14] Duque A, McCormick DA (2010). Circuit-based localization of ferret prefrontal cortex. Cereb Cortex.

[CR15] Elgueda D, Duque D, Radtke-Schuller S (2019). State-dependent encoding of sound and behavioral meaning in a tertiary region of the ferret auditory cortex. Nat Neurosci.

[CR16] Erickson SL, Lewis DA (2004). Cortical connections of the lateral mediodorsal thalamus in cynomolgus monkeys. J Comp Neurol.

[CR17] Fang P-C, Stepniewska I, Kaas JHH (2006). The thalamic connections of motor, premotor, and prefrontal areas of cortex in a prosimian primate (*Otolemur garnetti*). Neuroscience.

[CR18] Francis NA, Radtke-Schuller S, Fritz JB, Shamma SA (2018). Neural responses in dorsal prefrontal cortex reflect proactive interference during an auditory reversal task. Bioscience.

[CR19] Fritz JB, David SV, Radtke-Schuller S (2010). Adaptive, behaviorally gated, persistent encoding of task-relevant auditory information in ferret frontal cortex. Nat Neurosci.

[CR20] Fuster J (2015). The prefrontal cortex.

[CR21] Gallyas F (1979). Silver staining of myelin by means of physical development. Neurol Res.

[CR22] Goldman-Rakic PS, Porrino LJ (1985). The primate mediodorsal (MD) nucleus and its projection to the frontal lobe. J Comp Neurol.

[CR23] Guitton D, Mandl G, Medical A (1978). Frontal “oculomotor” area in alert cat. I. Eye movements and neck activity evoked by stimulation. Brain Res.

[CR24] Herbert J (1963). Nuclear structure of the thalamus of the ferret. J Comp Neurol.

[CR25] Hintzen A, Pelzer EA, Tittgemeyer M (2018). Thalamic interactions of cerebellum and basal ganglia. Brain Struct Funct.

[CR26] Hoover WB, Vertes RP (2007). Anatomical analysis of afferent projections to the medial prefrontal cortex in the rat. Brain Struct Funct.

[CR27] Jones EG (1985). The thalamus.

[CR28] Kandel ER, Schwartz JH (2000). Principles of neural science.

[CR29] Katayama T, Niimi K, Matsuoka H (1986). Thalamic projections to the motor and premotor areas in the cat traced with horseradish peroxidase. J Hirnforsch.

[CR30] Kirouac GJ (2015). Placing the paraventricular nucleus of the thalamus within the brain circuits that control behavior. Neurosci Biobehav Rev.

[CR31] Kosmal A (1981). Subcortical connections of the prefrontal cortex in dogs: afferents to the proreal gyrus. Acta Neurobiol Exp (Wars).

[CR32] Kosmal A (1981). Subcortical connections of the prefrontal cortex in dogs: afferents to the medial cortex. Acta Neurobiol Exp (Wars).

[CR33] Kosmal A (1986). Topographical organization of frontal association cortex afferents originating in ventral thalamic nuclei in dog brain. Ventral Thalamic Nuclei.

[CR34] Kosmal A, Dabrowska J (1980). Subcortical connections of the prefrontal cortex in dogs: afferents to the orbital gyrus. Acta Neurobiol Exp.

[CR89] Kreiner J (1961). The myeloarchitectonics of the frontal cortex of the dog. J Comp Neurol.

[CR35] Krimer LS, Goldman-Rakic PS (2001). Prefrontal microcircuits: membrane properties and excitatory input of local, medium, and wide arbor interneurons. J Neurosci.

[CR36] Kroenke CD, Mills BD, Olavarria JF, Neil JJ (2014). Neuroanatomy of the ferret brain with focus on the cerebral cortex. Biology and diseases of the ferret.

[CR37] Llewellyn-Smith IJ, Pilowsky P, Minson JB (1993). The tungstate-stabilized tetramethylbenzidine reaction for light and electron microscopic immunocytochemistry and for revealing biocytin-filled neurons. J Neurosci Methods.

[CR38] Mansouri FA, Koechlin E, Rosa MGP, Buckley MJ (2017). Managing competing goals–a key role for the frontopolar cortex. Nat Rev Neurosci.

[CR39] Markowitsch HJ, Pritzel M, Divac I (1978). The prefrontral cortex of the cat: anatomical subdivisions based on retrograde labeling of cells in the mediodorsal thalamic nucleus. Exp Brain Res.

[CR40] Martínez-Moreno E, Llamas A, Avendaño C (1987). General plan of the thalamic projections to the prefrontal cortex in the cat. Brain Res.

[CR41] Matelli M, Luppino G, Fogassi L, Rizzolatti G (1989). Thalamic input to inferior area six and area four in the macaque monkey. J Comp Neurol.

[CR42] McCormick DA, Shu Y, Hasenstaub A (2003). Persistent cortical activity: mechanisms of generation and effects on neuronal excitability. Cereb Cortex.

[CR43] Mesulam M-M (1978). Tetramethyl benzidine for horseradish peroxidase neurohistochemistry: a non-carcinogenic blue reaction- product with superior sensitivity for visualizing neural afferents and efferents. J Histochem Cytochem.

[CR44] Mitchell AS, Chakraborty S (2013). What does the mediodorsal thalamus do?. Front Syst Neurosci.

[CR45] Moran MA, Reinoso-Suárez F (1988). Topographical organization of the afferent connections to the motor cortex in the cat. J Comp Neurol.

[CR46] Morel A, Liu J, Wannier T (2005). Divergence and convergence of thalamocortical projections to premotor and supplementary motor cortex: a multiple tracing study in the macaque monkey. Eur J Neurosci.

[CR47] Narkiewicz O, Brutkowski S (1967). The organization of projections from the thalamic mediodorsal nucleus to the prefrontal cortex of the dog. J Comp Neurol.

[CR48] Nieuwenhuys R, Voogd J, Van Huijzen C (2008). The human central nervous system. the human central nervous system.

[CR49] nigel i, lawes c, andrews P (1998). Neuroanatomy of the ferret brain. biology and diseases of the ferret.

[CR50] olszewski j (1952). The thalamus of the macaca mulatta: an atlas for use with the stereotaxic instrument.

[CR51] perry B, mitchell as (2019). Considering the evidence for anterior and laterodorsal thalamic nuclei as higher order relays to cortex. front Mol Neurosci.

[CR52] Preuss TM (1995). Do rats have prefrontal cortex? The Rose-Woolsey-Akert program reconsidered. Cogn Neurosci J.

[CR53] Purves D (2004). Neuroscience.

[CR54] Radtke-Schuller S (2018). Cyto- and Myeloarchitectural Brain atlas of the ferret (Mustela putorius) in mri aided stereotaxic coordinates.

[CR55] Rajkowska G, Kosmal A (1988). Intrinsic connections and cytoarchitectonic data of the frontal association cortex in the dog. Acta Neurobiol EXP.

[CR56] Ray JP, Price JL (1993). The organization of projections from the mediodorsal nucleus of the thalamus to orbital and medial prefrontal cortex in macaque monkeys. J Comp Neurol.

[CR57] Ray JP, Price JL (1992). The organization of the thalamocortical connections of the mediodorsal thalamic nucleus in the rat, related to the ventral forebrain? Prefrontal cortex topography. J Comp Neurol.

[CR58] Rebollo B, Perez-Zabalza M, Ruiz-Mejias M (2018). Beta and gamma oscillations in prefrontal cortex during nmda hypofunction: an in vitro model of schizophrenia features. Neuroscience.

[CR59] Reep R (1984). Relationship between prefrontal and limbic cortex: a comparative anatomical review. Brain Behav Evol.

[CR60] Rinvik E (1968). The corticothalamic projection from the gyrus proreus and the medial wall of the rostral hemisphere in the cat an experimental study with silver impregnation methods. Exp brain Res.

[CR61] Romo R, de Lafuente V (2013). Conversion of sensory signals into perceptual decisions. Prog Neurobiol.

[CR62] Rosa MGP, Soares JGM, Chaplin TA (2019). Cortical afferents of area ten in cebus monkeys: implications for the evolution of the frontal pole. Cereb Cortex.

[CR63] Rose JE, Woolsey CN (1948). The orbitofrontal cortex and its connections with the mediodorsal nucleus in rabbit, sheep and cat. Res Publ Assoc Res Nerv Ment Dis.

[CR64] Saalmann YB (2014). Intralaminar and medial thalamic influence on cortical synchrony, information transmission and cognition. Front Syst Neurosci.

[CR65] Sakai ST, Stanton GB, Isaacson LG (1993). Thalamic afferents of area four and six in the dog: a multiple retrograde fluorescent dye study. Anat Embryol.

[CR66] Schell GR, Strick PL (1984). The origin of thalamic inputs to the arcuate premotor and supplementary motor areas. J Neurosci.

[CR67] Schlag J, Schlag-Rey M (1970). Induction of oculomotor responses by electrical stimulation of the prefrontal cortex in the cat. Brain Res.

[CR68] Sellers KK, Bennett DV, Hutt A (2015). Awake vs. anesthetized: layer-specific sensory processing in visual cortex and functional connectivity between cortical areas. J Neurophysiol.

[CR69] Sellers KK, Bennett DV, Hutt A, Fröhlich F (2013). Anesthesia differentially modulates spontaneous network dynamics by cortical area and layer. J Neurophysiol.

[CR70] Sellers KK, Yu C, Zhou ZC (2016). Oscillatory dynamics in the frontoparietal attention network during sustained attention in the ferret. Cell Rep.

[CR71] Shu Y, Duque A, Yu Y (2007). Properties of action-potential initiation in neocortical pyramidal cells: evidence from whole cell axon recordings. J Neurophysiol.

[CR72] Shu Y, Hasenstaub A, Duque A (2006). Modulation of intracortical synaptic potentials by presynaptic somatic membrane potential. Nature.

[CR73] Stanton GB, Tanaka D, Sakai ST, Weeks OI (1986). Thalamic afferents to cytoarchitectonic subdivisions of area six on the anterior sigmoid gyrus of the dog: a retrograde and anterograde tracing study. J Comp Neurol.

[CR74] Stepniewska I, Preuss TMM, Kaas JHH (2007). Thalamic connections of the dorsal and ventral premotor areas in New World owl monkeys. Neuroscience.

[CR75] Tanaka D (1977). Projections from orbitofrontal cortex to mediodorsal thalamic nucleus in the dog. Brain Res.

[CR76] Uylings HBM, Groenewegen HJ, Kolb B (2003). Do rats have a prefrontal cortex?. Behav Brain Res.

[CR77] Van Der Werf YD, Witter MP, Groenewegen HJ (2002). The intralaminar and midline nuclei of the thalamus. Anatomical and functional evidence for participation in processes of arousal and awareness. Brain Res Rev.

[CR78] Varela C, Kumar S, Yang JY, Wilson MA (2014). Anatomical substrates for direct interactions between hippocampus, medial prefrontal cortex, and the thalamic nucleus reuniens. Brain Struct Funct.

[CR79] Vedovato M (1978). Identification of afferent connections to cortical area 6aβ of the cat by means of retrograde horseradish peroxidase transport. Neurosci Lett.

[CR80] Vertes RP, Hoover WB (2008). Projections of the paraventricular and paratenial nuclei of the dorsal midline thalamus in the rat. J Comp Neurol.

[CR81] Vertes RP, Linley SB, Hoover WB (2015). Limbic circuitry of the midline thalamus. Neurosci Biobehav Rev.

[CR82] Walker AE (1940). A cytoarchitectural study of the prefrontal area of the macaque monkey. J Comp Neurol.

[CR83] Wang Y, Markram H, Goodman PH (2006). Heterogeneity in the pyramidal network of the medial prefrontal cortex. Nat Neurosci.

[CR85] Winograd M, Destexhe A, Sanchez-Vives MV (2008). Hyperpolarization-activated graded persistent activity in the prefrontal cortex. Proc Natl Acad Sci.

[CR86] Wise SP (2008). Forward frontal fields: phylogeny and fundamental function. Trends Neurosci.

[CR87] Wollstadt P, Sellers KK, Rudelt L (2017). Breakdown of local information processing may underlie isoflurane anesthesia effects. PLoS Comput Biol.

[CR88] Zhou ZC, Yu C, Sellers KK, Fröhlich F (2016). Dorso-lateral frontal cortex of the ferret encodes perceptual difficulty during visual discrimination. Sci Rep.

